# Engineering cascade biocatalysis in whole cells for bottom-up synthesis of cello-oligosaccharides: flux control over three enzymatic steps enables soluble production

**DOI:** 10.1186/s12934-022-01781-w

**Published:** 2022-04-09

**Authors:** Katharina N. Schwaiger, Alena Voit, Birgit Wiltschi, Bernd Nidetzky

**Affiliations:** 1 ACIB-Austrian Centre of Industrial Biotechnology, Krenngasse 37, 8010 Graz, Austria; 2 Institute of Biotechnology and Biochemical Engineering, NAWI Graz, Graz University of Technology, Petersgasse 12, 8010 Graz, Austria

**Keywords:** Cello-oligosaccharides, Multi-enzymatic cascade, Whole-cell catalyst, Co-expression, Activity ratios

## Abstract

**Background:**

Soluble cello-oligosaccharides (COS, β‐1,4‐D‐gluco‐oligosaccharides with degree of polymerization DP 2–6) have been receiving increased attention in different industrial sectors, from food and feed to cosmetics. Development of large-scale COS applications requires cost-effective technologies for their production. Cascade biocatalysis by the three enzymes sucrose-, cellobiose- and cellodextrin phosphorylase is promising because it enables bottom-up synthesis of COS from expedient substrates such as sucrose and glucose. A whole-cell-derived catalyst that incorporates the required enzyme activities from suitable co-expression would represent an important step towards making the cascade reaction fit for production. Multi-enzyme co-expression to reach distinct activity ratios is challenging in general, but it requires special emphasis for the synthesis of COS. Only a finely tuned balance between formation and elongation of the oligosaccharide precursor cellobiose results in the desired COS.

**Results:**

Here, we show the integration of cellodextrin phosphorylase into a cellobiose-producing whole-cell catalyst. We arranged the co-expression cassettes such that their expression levels were upregulated. The most effective strategy involved a custom vector design that placed the coding sequences for cellobiose phosphorylase (CbP), cellodextrin phosphorylase (CdP) and sucrose phosphorylase (ScP) in a tricistron in the given order. The expression of the tricistron was controlled by the strong T7_*lacO*_ promoter and strong ribosome binding sites (RBS) for each open reading frame. The resulting whole-cell catalyst achieved a recombinant protein yield of 46% of total intracellular protein in an optimal ScP:CbP:CdP activity ratio of 10:2.9:0.6, yielding an overall activity of 315 U/g dry cell mass. We demonstrated that bioconversion catalyzed by a semi-permeabilized whole-cell catalyst achieved an industrial relevant COS product titer of 125 g/L and a space–time yield of 20 g/L/h. With CbP as the cellobiose providing enzyme, flux into higher oligosaccharides (DP ≥ 6) was prevented and no insoluble products were formed after 6 h of conversion.

**Conclusions:**

A whole-cell catalyst for COS biosynthesis was developed. The coordinated co-expression of the three biosynthesis enzymes balanced the activities of the individual enzymes such that COS production was maximized. With the flux control set to minimize the share of insolubles in the product, the whole-cell synthesis shows a performance with respect to yield, productivity, product concentration and quality that is promising for industrial production.

**Supplementary Information:**

The online version contains supplementary material available at 10.1186/s12934-022-01781-w.

## Background

Cello-oligosaccharides (COS) are linear β-1,4-linked gluco-oligosaccharides with promising functional properties and broad applicability. Soluble COS with a degree of polymerization (DP) from 2 to 6 (G2-G6) are particularly interesting. Their prebiotic function [1–5] is most auspicious for medicine, but also attracts the animal feed industry [3–5]. COS inhibit growth of pathogenic microorganisms, such as *Clostridium* sp. [1, 6], and lower the total cholesterol and neutral fat concentration in the liver when orally ingested [1, 7]. COS are low-calory sweeteners and can be applied as a bulking agent in the food industry [1, 7]. Their particular physico-chemical, mechanical and rheological properties, such as resistance to compression, malleability, heat and acidic pH, could facilitate their incorporation in food formulations, dietary supplements or medications [1]. Furthermore, COS show considerable potential for the cosmetics industry: Their moisturizing effect and ability to inhibit growth of pathogenic bacteria (e.g. *Staphylococcus* sp.) lead to an improved skin barrier [1, 7].

To develop industrial applications of the COS, they must be produced efficiently at large scale. The reported synthetic routes to COS, however, involve challenges. Synthesis can be either top-down from cellulose or bottom-up from suitable precursors for oligomerization (e.g., glucose, cellobiose). The top-down synthesis from cellulose either needs harsh conditions for the polysaccharide chains to be hydrolysed chemically or it requires a well-investigated cellulolytic enzyme cocktail [1, 8–10]. Moreover, uncontrolled enzymatic depolymerization exclusively leads to glucose and cellobiose [8]. The use of a cellulase mixture without glucosidases requires a complicated and expensive enzyme separation procedure, hampering its application on a large scale [11].

**Fig. 1:**
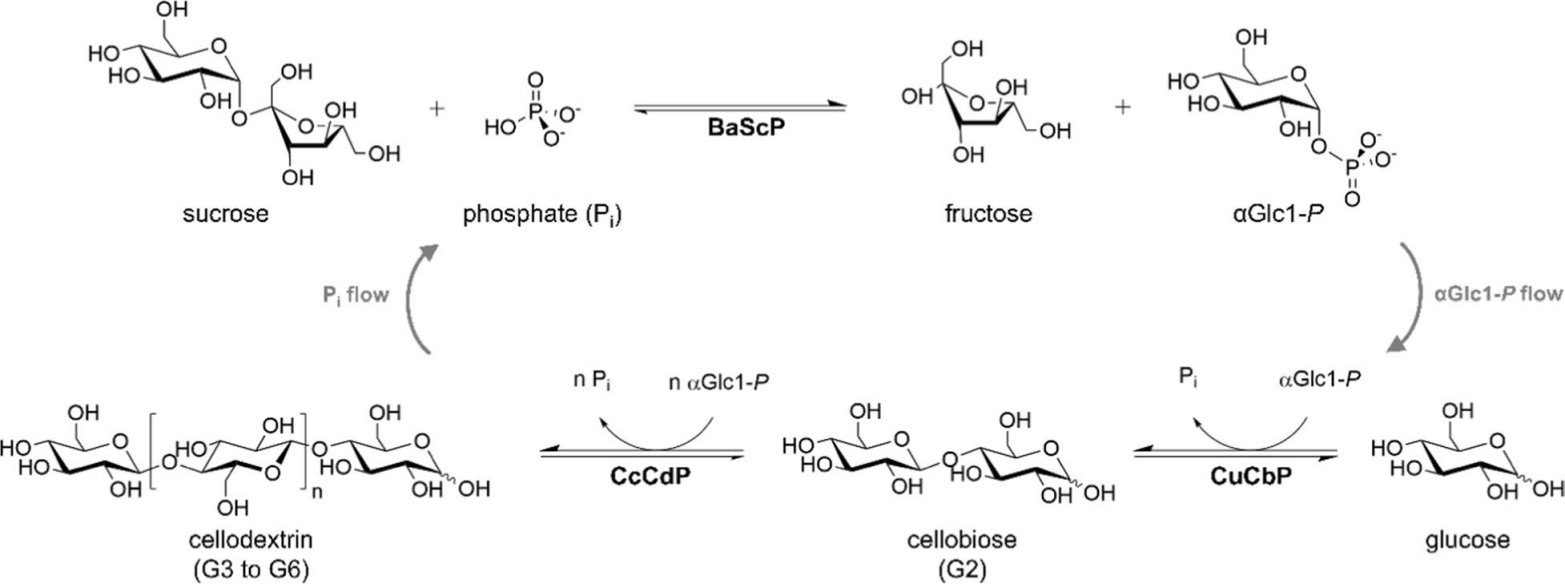
Enzymatic production of COS using sucrose phosphorylase (BaScP), cellobiose phosphorylase (CuCbP) and cellodextrin phosphorylase (CcCdP). For soluble COS production (DP ≤ 6), *n* is 1 to 4

Current approaches of bottom-up synthesis of COS by cellulases [8, 12] or glycosynthases [8, 13] rely on non-natural (synthetic) glycosyl donors, e.g., cellobiose fluoride, and typically end up with oligomers modified (e.g., 4-*O*-methyl) at the non-reducing end. Sugar nucleotide-dependent glycosyltransferases were shown to perform iterative β-1,4-glycosylation from an activated donor substrate, e.g., UDP-glucose [14]. The UDP-glucose might be prepared from an expedient donor such as sucrose [15], but applicability of glycosyltransferase cascade reactions to COS synthesis remains to be demonstrated. By contrast, cascade reactions of glycoside phosphorylases are well-developed and provide a promising route to COS [2, 16–28]. The development of a three-enzyme cascade (Fig. 1) circumvents the requirement of the expensive phosphorylase donor substrate α-glucose 1-phosphate (αGlc1-*P*). The reaction starts from the inexpensive sugars sucrose and glucose [2, 24, 25, 28]. Sucrose phosphorylase (from *Bifidobacterium adolescentis*, BaScP) converts sucrose and phosphate to αGlc1-*P* and fructose in the first step. Cellobiose phosphorylase (from *Cellulomonas uda*, CuCbP) and cellodextrin phosphorylase (from *Clostridium cellulosi*, CcCdP) further use αGlc1-*P* for oligomerization by iterative β-1,4-glycosylation (Fig. 1). Bottom-up synthesis by phosphorylases in the way described in Fig. 1 gives COS with a DP distribution centered at 3–5. The conversion was optimized towards COS concentrations of up to 93 g/L and scaled up to COS production in low g amounts [2].

DP control in the oligosaccharide product is the crucial element of successful COS production. In the absence of such control, the CdP continues the β-1,4-chain oligomerization to a point (DP ≥ 6) at which self-assembly driven aggregation of the oligosaccharides from solution can no longer be suppressed adequately [17, 23, 29, 30]. While useful for the preparation of insoluble cellulose with property-tunable characteristics of material [13, 25, 29, 31–35], product precipitation lowers the efficiency of the COS production. DP control can be achieved by restraining the activity of the CdP relative to the activities of ScP and CbP to maximize the flux through the three steps of the reaction cascade. It was shown previously that purified BaScP, CuCbP and CcCdP enzymes at a relative activity ratio of 10:3:2 catalyze COS synthesis without the formation of insoluble product [2, 24]. However, distinct activity ratios can easily be achieved when separate enzyme formulations are used. Nonetheless, the production of each individual enzyme is time-consuming and generates waste, as shown with other enzymes by Woodley et al.: Crude and isolated enzyme preparations are two- and ten-fold more costly, respectively, than the corresponding whole-cell preparation [36]. The economic efficiency of the whole-cell approach, therefore increases sharply with the number of enzymes involved in the cascade reaction. The current study was performed to develop a three-enzyme whole-cell catalyst for COS production.

Here, a precise co-expression strategy is especially important, firstly because phosphorylase enzymes differ substantially in specific activities (up to 30-fold), and secondly because the CdP-catalyzed over-elongation should be avoided. Optimization of mismatching enzyme activities on the expression level has been reported sparsely in the literature [37–39] and is primarily limited to two-enzyme systems [40–44]. Co-expression strategies in *Escherichia coli* are frequently built upon the commercial pETDuet vector, which harbors two expression cassettes, each controlled by a T7_*lacO*_ promoter. Finely tuned balancing of enzyme activities, however, requires further engineering efforts often targeted at downregulation of strong control elements, e.g., promoters, the ribosome binding sites, or both [39, 43, 45], thereby reducing the overall recombinant protein level.

Employing a new strategy of whole-cell engineering for cascade biocatalysis, here we focused on upregulating, rather than downregulating the individual enzyme expressions to fine-tune their relative activities in an *E. coli*-derived whole-cell catalyst. In addition to an optimal activity ratio, the catalytic efficiency of a cell catalyst also depends on the overall recombinant protein yield. Therefore, we avoided using downregulatory expression strategies, where cutbacks in protein yields would have to be compensated by using higher amounts of biomass in the bioconversion. Previously, we demonstrated the successful whole-cell production of cellobiose by the tuned co-expression of BaScP and CuCbP [40]. The best performing plasmid from this study, pBICI_2_strong (further referred to as pBICI for simplicity), represented the starting point for the three-enzyme co-expression strategies pursued in this work. pBICI facilitates the bicistronic expression of the CuCbP and BaScP coding sequences in this order downstream of the T7_*lacO*_ promoter. To produce COS in the current study, we integrated CcCdP without reducing already achieved CuCbP and BaScP activities expressed from pBICI by gene order rearrangements of the expression system. The resulting three-enzyme whole-cell catalysts were compared in terms of COS yield and product composition (DP 2–6). Additionally, we evaluated cell permeabilization and reaction conditions, such as temperature and substrate concentration, for the optimization of the bioconversion. We achieved the biosynthesis of COS at a level that can be interesting for industrial production.

## Results and discussion

The cascade reaction according to Zhong et al. [24] was considered for COS synthesis (Fig. 1). Of the three enzymes used in whole-cell catalyst development, the BaScP (GenBank identifier AF543301.1) was most active (117 ± 10 U/mg; *N* = 6), followed by CuCbP (11.4 ± 0.3 U/mg; *N* = 3; GenBank identifier AAQ20920.1) and CcCdP (4.4 ± 0.5 U/mg; *N* = 6; GenBank identifier CDZ24361.1). The specific activities of the purified enzymes are from this study and were assayed at 30 °C.

**Fig. 2:**
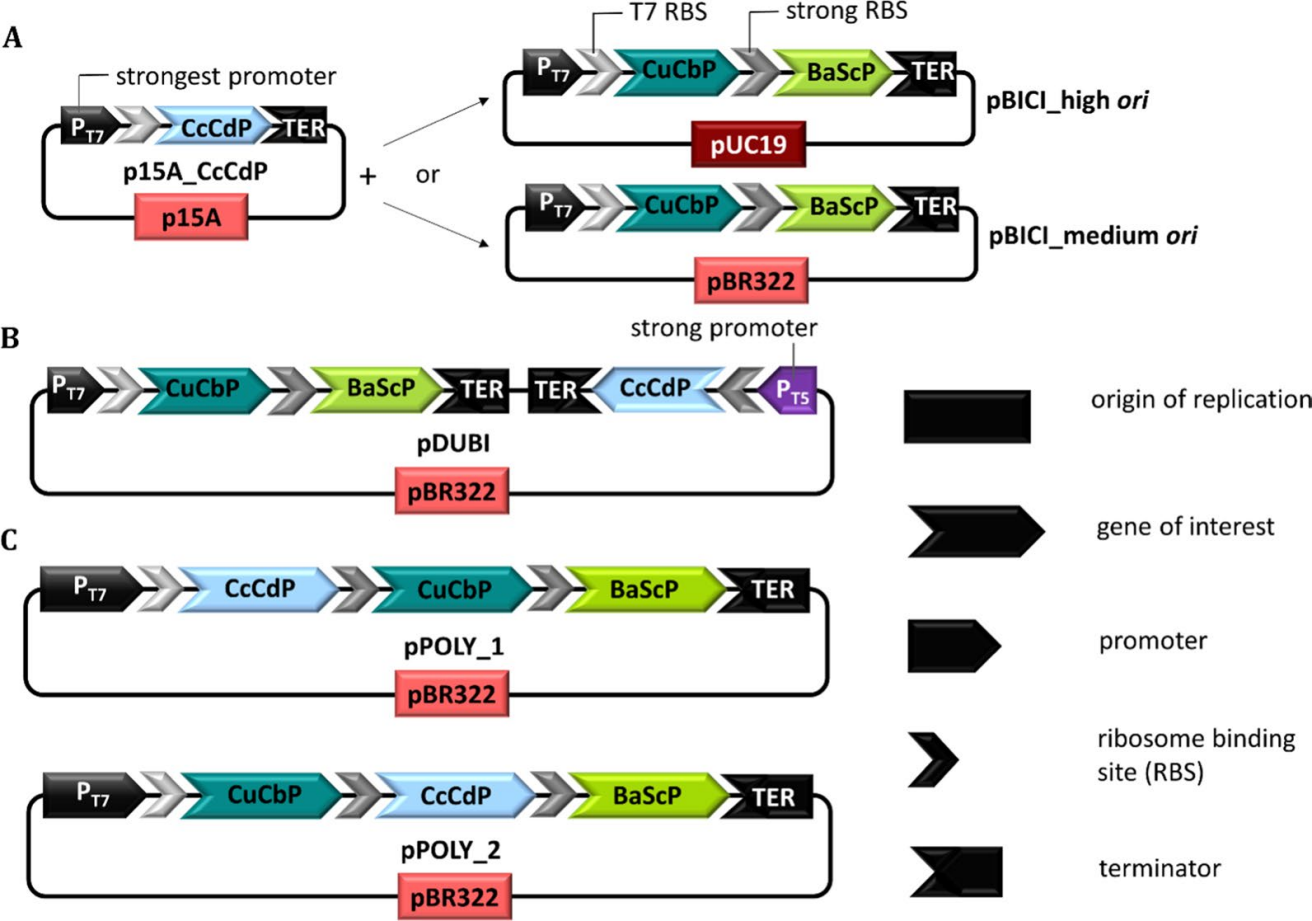
Vector design for enzyme co-expression with two plasmids. **A** p15A and pBR322 medium copy *ori*, pUC19 high copy *ori*. Single plasmid co-expression: Combinatorial strategy (**B** pDUBI) with two expression cassettes oriented in opposite directions and polycistronic strategies (**C** pPOLY_1 and pPOLY_2). All plasmid maps are depicted in full in the Additional file 1: Fig. S3. Additionally, the plasmid maps and full sequences were deposited in the add gene database, accession numbers 179272 to 179276 (https://www.addgene.org)

The custom plasmid vector pBICI (corresponds to pBICI_2_strong in [40]) was used for co-expression of CuCbP and BaScP. The plasmid is represented in Fig. 2A (here: pBICI_medium *ori*) and full details are given in the addgene plasmid database (https://www.addgene.org/155168/). Based on specific activities of BaScP (13.9 U/mg) and CuCbP (3.0 U/mg) in the *E. coli* cell extract, the enzyme activity ratio (BaScP:CuCbP) was ~ 4.7 [40]. The previously reported results were confirmed in this study and served as point of reference for the co-expression of CcCdP with CuCbP and BaScP.

### CcCdP integration strategies

#### Co-expression based on two plasmids leads to plasmid modification

In a first approach, we co-expressed the three biosynthesis enzymes from two plasmids. CcCdP was expressed from plasmid vector p15A (Fig. 2A). p15A was small in size and the design allowed its co-existence with plasmid pBICI in *E. coli* due to compatible origins of replication and antibiotic resistance markers. It was assembled synthetically from in house available DNA parts (Additional file 1: Table S1). As shown in Fig. 2A, plasmid pBICI was used in its original design with the medium-copy pBR322 origin of replication (*ori*) and as a multi-copy version carrying the high-copy *ori* pUC19 (pBICI_high *ori*). A glucose-1-phosphatase deficient expression strain *E. coli* BL21(DE3)*agp*^–^ (obtained from Tom Desmet, Ghent University, Belgium) was used to prevent degradation of αGlc1-*P* (Fig. 1) in whole-cell conversions. This strain was co-transformed with plasmid p15A and the relevant pBICI vectors. The plasmids were maintained by supplementing the expression cultures with kanamycin and ampicillin (for details see  "Cell preparation" sub section in the "Methods" section). Extract from lysed cells was used for activity determination.

**Fig. 3:**
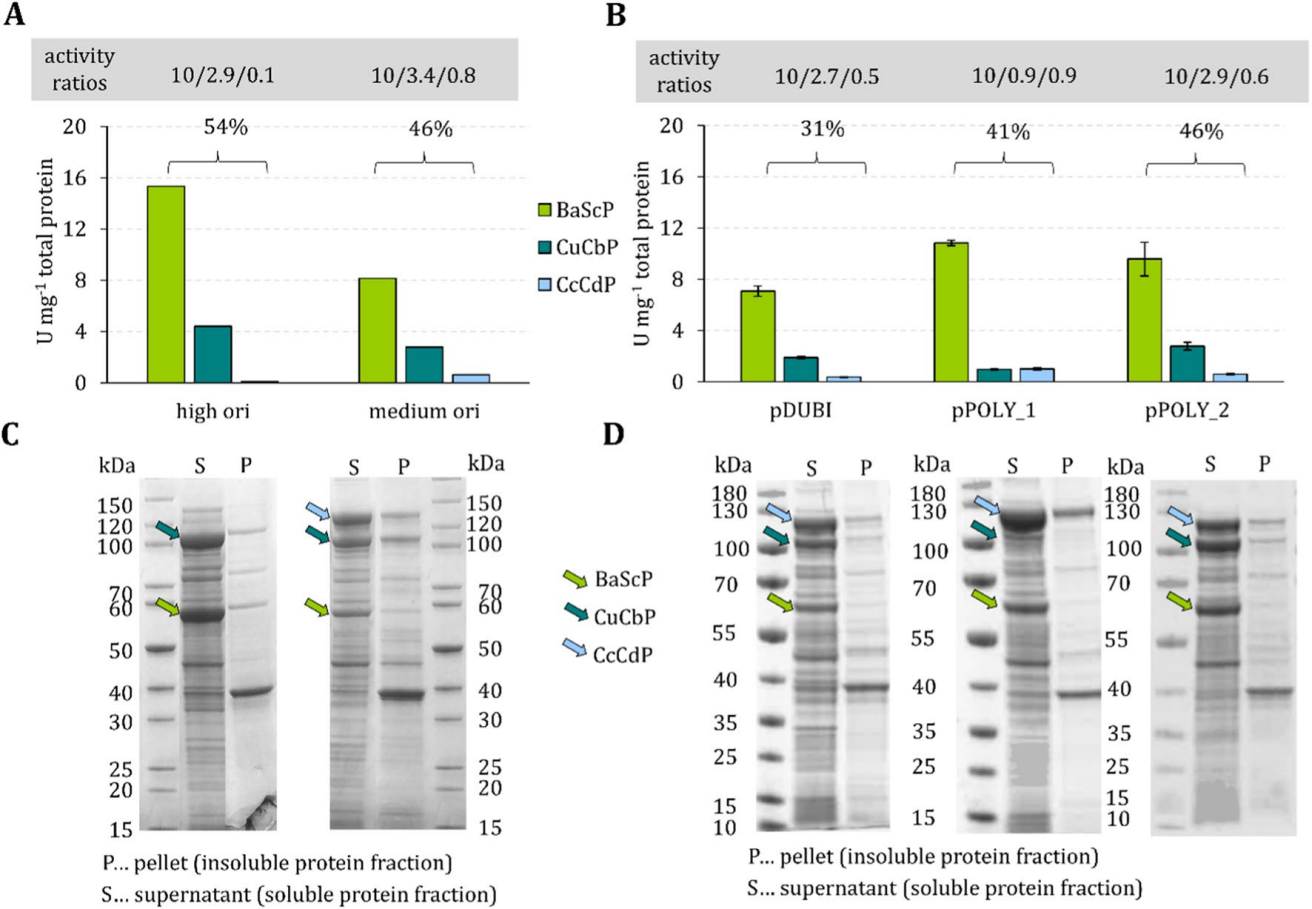
Expression performance of two-plasmid (**A, C**) and single-plasmid (**B, D**) co-expression strategies. Comparison of specific activities (synthesis) in cell-free extracts, relative activity ratios normalized to BaScP activity and percentage share of recombinant protein yields; **A** one expression each; **B** three expressions each, standard deviations are shown as error bars. Representative SDS polyacrylamide gels are depicted in **C** and **D**

Plasmid p15A used together with pBICI_high *ori* resulted in only low CcCdP activity (0.1 U/mg). In contrast, the activities of BaScP (15.3 U/mg) and CuCbP (4.4 U/mg) were high, as shown in Fig. 3A,C. Carrying a multi-copy origin of replication, pBICI_high *ori* (pUC19 *ori*, several hundreds of copies per cell [46]) was expected to exceed p15A (p15A *ori,* ~ 20 copies/cell [46]) in copy number by roughly one order of magnitude. We estimated the abundance of the enzymes in the cell extract by comparing their specific activities in cell extract to the isolated enzymes. Taken together, BaScP (13%) and CuCbP (39%) accounted for ~ 52% of the total soluble *E. coli* protein (Fig. 3A). The low production of CcCdP (2%) might be explained by a limited allocation of cellular resources for gene expression from plasmid p15A compared to pBICI_high *ori*, if the latter was present in excess. Cell resource limitations at high plasmid copy numbers [47–49] and the imbalanced distribution of scant resources among co-existing plasmids [43] were reported previously in the literature.

Plasmid p15A used together with pBICI_medium *ori* gave a promising set of specific activities for BaScP (8.2 U/mg), CuCbP (2.8 U/mg) and CcCdP (0.6 U/mg; Fig. 3A). From their respective *ori*, we expected the two plasmids to be maintained at similar copy numbers of ~ 20 [46]. Compared to enzyme co-expression involving pBICI_high *ori*, the activities of BaScP and CuCbP were decreased ~ 1.9- and 1.6-fold while the activity of CcCdP was increased sevenfold. These results would be consistent with the idea that because of plasmid copy number, pBICI_high *ori* outcompetes p15A in respect to the allocation of cell resources. However, although the exact same plasmid p15A was used in combination with pBICI_high *ori* and pBICI_medium *ori*, the interpretation of the results must consider the possibility that the p15A copy number varies in dependence of the other plasmid's copy number (see ref. [43]).

Serial co-expressions using p15A in combination with pBICI_medium *ori* revealed a rapid decline in the expression of BaScP and CuCbP while the CcCdP continued to be fully expressed. Figure S1 (Additional file 1) shows that already in the second round of co-expression, BaScP and CuCbP were no longer detectable by SDS-PAGE analysis of the cell extract. Restriction analysis of the re-isolated plasmids revealed pBICI_medium *ori* to have been truncated by ~ 4.000 bp (Additional file 1: Figure S1). Plasmid p15A was intact. Considering modifications of the pBICI vector noted previously [40], the truncation of pBICI_medium *ori* observed in this study may have involved excision of the BaScP and CuCbP genes. Low operational stability was a major disadvantage of the two-plasmid co-expression approach. Requirement for two antibiotics complicates the cell cultivation and presents an additional hurdle for process scale up to production. Therefore, we sought alternative strategies for the co-expression of the three phosphorylases.

#### Single-plasmid co-expression strategies: a tricistronic expression system outperforms a hybrid plasmid vector

The co-expression of three enzymes from a single plasmid comprised two strategies: A combinatorial expression system, involving two separate promoters (pDUBI, Fig. 2B); and a tricistronic expression vector (pPOLY, Fig. 2C). Both plasmids originated from pBICI medium *ori*. To achieve high expression levels of CcCdP and, thus, compensate for its low specific activity (4.4 ± 0.5 U/mg), its coding sequence was integrated into pBICI medium *ori* under control of the strong promoter T5_*lacO*_ [50], resulting in pDUBI (Fig. 2B). Expression of BaScP and CuCbP used the reported bicistronic design [40] under control of the promoter T7_*lacO*_. The two separate expression cassettes were oriented in opposite directions. Secondly, we integrated the CcCdP coding sequence into the T7_*lacO*_-BaScP-CuCbP bicistron on pBICI medium *ori*, resulting in a tricistronic expression cassette (pPOLY). This system allowed us to address mismatching enzyme activities by rearranging the order of coding sequences in the tricistron, thus exploiting the gene-order dependence of expression [51, 52]. Aiming for high or moderate expression levels, we positioned CcCdP at the first (pPOLY_1) or second position (pPOLY_2) in the tricistron (Fig. 2C).

We characterized the expression of the individual genes in three consecutively cultivated biological replicates. Specific activity ratios in cell-free extracts (BaScP:CuCbP:CcCdP) were 7.1:1.9:0.4 U/mg for pDUBI, 10.8:1.0:1.0 U/mg for pPOLY_1 and 9.6:2.8:0.6 U/mg for pPOLY_2. Variation coefficients from three independent biological replicates were below 14% for each enzyme activity and plasmid. The fractions of recombinant BaScP, CuCbP and CcCdP in total soluble *E. coli* protein were 31%, 41% and 46% for pDUBI, pPOLY_1 and pPOLY_2, respectively (Fig. 3B, D). As expected, expression with pPOLY_1 resulted in higher CcCdP activities than the T5_*lacO*_ controlled expression (pDUBI) as well as expression from position two in the tricistron (pPOLY_2). Using pPOLY_1, CcCdP and CuCbP were expressed at equal activities (Fig. 3B). In comparison, expression with pDUBI and pPOLY_2 resulted in a ~ fivefold higher activity of CuCbP than CcCdP. The relative activity ratios (normalized to BaScP activity) obtained with pDUBI and pPOLY_2 were comparable to one another, i.e., 10:2.7:0.5 and 10:2.9:0.6 (Fig. 3B). However, pPOLY_2 outperformed pDUBI and pPOLY_1 in terms of the overall recombinant protein yield (46%, Fig. 3B). Tentatively, the lower overall recombinant protein expression level with pDUBI might derive from pausing and backtracking of RNA-polymerases as a result of increased RNA-polymerase traffic on the plasmid caused by the presence of two strong promoters [53]. A lower copy number of pDUBI compared to the two pPOLY plasmids seems unlikely. Plasmid size can affect copy number by way of size dependence of the length of the plasmid replication phase [54]. However, pDUBI is negligibly larger (117 bp) than the pPOLY plasmids.

To assess potential effects of cultivation conditions on the expression performance of the cell catalysts carrying pPOLY_1, pPOLY_2 and pDUBI, we varied the isopropyl β-D-1-thiogalactopyranoside (IPTG) inductor concentration and the time post-induction (Additional file 1: Fig. S2A). The temperature was also varied during gene expression from pPOLY_2 (Additional file 1: Fig. S2B). However, as shown by SDS-PAGE, the expression levels were hardly changed compared to the benchmark condition (1.0 mM IPTG, 25 °C, 18 h; Additional file 1: Fig. S2). Recombinant gene expression from pPOLY_2 amounted to 46% of the total cellular protein, which was close to the upper limit of recombinant protein expression in *E. coli* of about ~ 50% [55–57].

### Cellodextrin synthesis depends on the whole-cell catalyst used

COS synthesis was performed with single-plasmid whole-cell catalysts using conditions according to Zhong et al. [24]. Reactions were not executed in biological replicates due to the low variation coefficients (< 14%) obtained from the expression study (see part *Single plasmid co-expression strategies*). The cell suspensions were frozen at -70 °C, thawed and added to 50 mL of reaction mixture at a concentration of 2.6 g_CDW_/L. The activities (in U/mL) measured from cell-free extracts are listed in Additional file 1: Table S2. Reactions were performed for 25 h at 30 °C. Full time-courses are depicted in Additional file 1: Fig. S4. Performance metrics such as product yield, product titre, space–time yield (STY) and total turnover number (TTN) (Table 1) were determined for the produced soluble COS (DP 2–6) after 8 h. The overall cell catalyst activity (Table 1) was calculated from the transferred glucosyl-units (αGlc1-*P*) to soluble COS (DP 2–6) after 1 h reaction time. The sum of glucosyl units was obtained as ∑c_*COS(DP)*_ × (DP-1), where c_*COS(DP)*_ is the concentration of the individual soluble COS of a certain DP.

**Table 1:** Performance metrics of freeze–thaw permeabilized cell catalysts in COS synthesis

	Soluble COS yield (8 h) %	Initial cell catalyst activity (1 h) U/g_CDW_	Product titer (8 h) g/L	STY (8 h) g/L/h	TTN (8 h) G_product_/g_CDW_
pPOLY_2	83	315	27	3.4	10.3
pPOLY_1	60	279	24	3.0	9.0
pDUBI	78	221	26	3.3	9.9

Reaction mixture consisted of 200 mM sucrose, 65 mM glucose, 50 mM phosphate, 50 mM MES, pH 7.0, 2.6 g_CDW_/L whole-cell catalyst

**Fig. 4:**
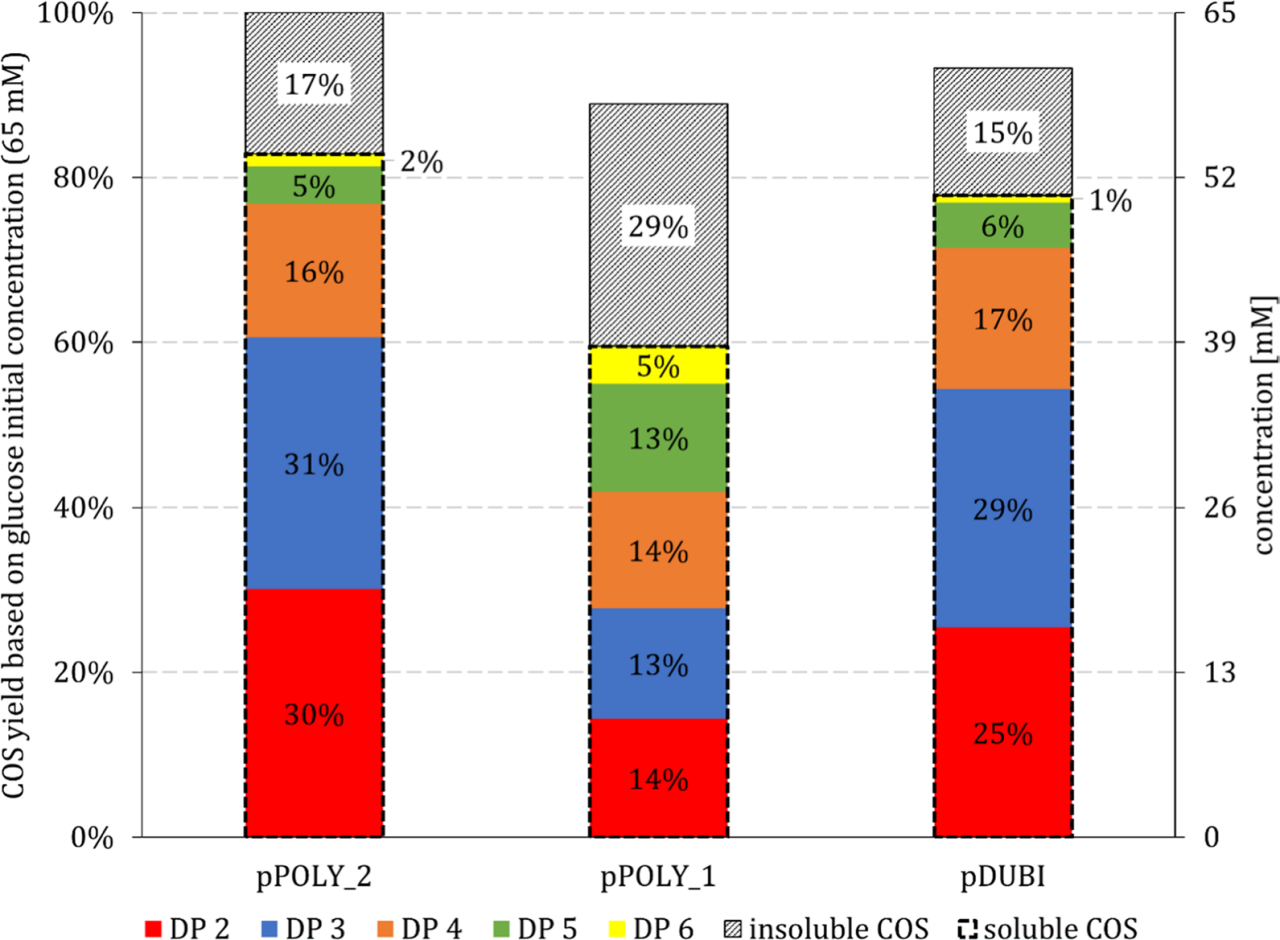
Product composition of single plasmid-based whole-cell catalysts after 8 h of conversion. Soluble COS are separated by their degree of polymerization (DP 2–6). Insoluble COS production was calculated based on the molar balance between glucose units incorporated as primer in soluble COS and the actually consumed glucose units

The whole-cell catalyst pPOLY_2 produced the highest COS titer of 27 g/L (DP 2–6; Table 1). Using free enzymes, Zhong et al. [24] had reported a higher product concentration of 39 g/L (DP 3–6). In their study they had performed the synthesis reaction with isolated enzymes at 45 °C while the temperature for the whole-cell-catalyzed conversion in the current study was 30 °C. The enzyme activity ratio used in Zhong et al. [24] was 10:3:2 (each U/mL) while it was 15:4:1 (each U/mL; Additional file 1: Table S2) here using the pPOLY_2 catalyst. The pDUBI catalyst yielded a similar product concentration but showed lower enzyme activities (10:3:0.5, each U/mL) than the pPOLY_2 catalyst. Since all three whole-cell catalysts achieved nearly full conversion of the substrate, the initial catalyst activity (calculated after 1-h reaction time) is the most suitable parameter for comparison. Here, the pPOLY_2 catalyst showed a 1.5-fold higher activity than the pDUBI catalyst (Table 1; 315 vs. 221 U/g_CDW_), while the pPOLY_1 catalyst was active at an intermediate level. With the pPOLY_1 catalyst, considerable amounts of insoluble COS were formed (29%; Fig. 4). Note that the insoluble product release was not included in the calculation of the activity in Table 1.

The substantial degree of insoluble COS formation in the reaction of the pPOLY_1 catalyst suggested a unique distribution of the individual enzymatic rates of the overall cascade transformation for this catalyst, different from the other two whole-cell catalysts. A relatively higher rate of oligomerization, as observed for the pPOLY_1 catalyst, would be expected to also affect the DP distribution of the soluble COS by the formation of a greater amount of longer-chain COS. Indeed, the COS produced by the pPOLY_1 catalyst exhibited an evener distribution of DP (13–14%; DP 2–5) than the COS produced by the pPOLY_2 and pDUBI catalysts (Fig. 4). The pPOLY_2 and pDUBI catalysts produced COS mixtures of similar composition, in accordance with their shared CuCbP:CcCdP ratio (~ 2.9:0.6 and ~ 2.7:0.5; Fig. 3B). The pPOLY_1 catalyst, conversely, showed a CuCbP:CcCdP ratio of ~ 1.0:1.0 (Fig. 3B). The relatively lower CuCbP activity can restrict the supply of cellobiose to the reaction of CcCdP and thus favors oligomerization towards higher DP COS, as shown in earlier work done with free enzymes [25]. Rate limitation by the CuCbP activity can explain the enhanced formation of insoluble product in the conversions by the pPOLY_1 catalyst.

In summary, the pPOLY_2 catalyst was most promising for soluble COS production. It outperformed the pPOLY_1 and pDUBI catalysts with regard to COS yield and conversion efficiency.

### Optimized COS synthesis using the pPOLY_2 whole-cell catalyst

**Fig. 5:**
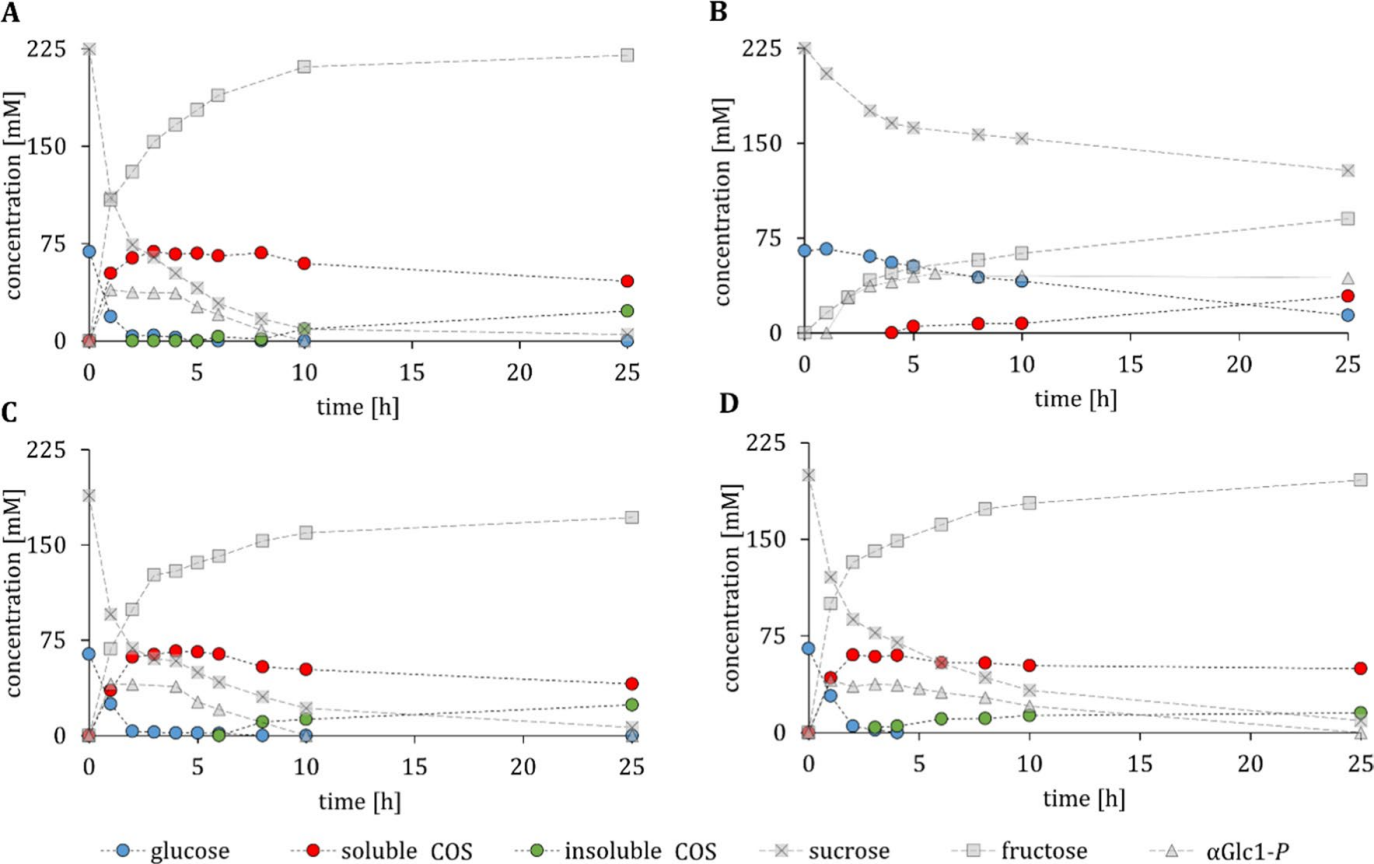
Different preparations of pPOLY_2 catalyst analyzed for COS synthesis: **A** cell-free extract, **B** non-permeabilized cells, **C** freeze-dried cells, **D** freeze–thaw treated cells. Reaction conditions: 200 mM sucrose, 65 mM glucose, 50 mM phosphate in 50 mM MES buffer, pH 7 at 30 °C, 2.6 g_CDW_/L reaction mixture

Whole-cell conversions are often limited by transport into and out of the cell [58]. The natural barrier function of the cellular membrane restricts the accessibility of the substrates to intracellular enzymes and might also prevent the release of products [58], particularly of high molecular weight products. To address a possible transport limitation, we tested two mechanical permeabilizations (freeze-drying and freeze-thawing) in conversions catalyzed by the pPOLY_2 catalyst. Both techniques are considered mild and maintain the whole-cell integrity [58, 59]. A cell-free extract preparation and non-permeabilized cells were used as positive and negative controls, respectively. The cell-free extract reached a soluble COS yield of 88% (Fig. 5A, Additional file 1: Table S3), whereas non-permeabilized cells reached only 11% (Fig. 5B; Additional file 1: Table S3). Conversions catalyzed by freeze-dried cells (85% soluble COS, Fig. 5C) and freeze–thaw treated cells (83% soluble COS, Fig. 5D) closely resembled the positive control. Enzyme leakage from the cells to the supernatant was lowest for freeze–thaw treated cells (~ 4.5% of the total recombinant protein; Additional file 1: Table S4). These results show that a single freeze–thaw cycle was very effective in permeabilizing the cells: The resulting catalyst was permissive for substrate/product transport but it restricted the enzyme release. Thus, the freeze–thaw treated catalyst showed good potential for recycling and was the preferred candidate for use in COS synthesis.

The conversions depicted in Fig. 5 were performed at 30 °C. The temperature was chosen to preserve the overall structural integrity as well as the enzyme activity of the whole-cell catalyst. Nevertheless, a reaction temperature increase to 45 °C, as suggested by Zhong et al. [24] in their study of the soluble enzymes, was also possible with the whole-cell catalyst. A temperature shift from 30 °C to 45 °C increased the STY by a remarkable 7.4-fold, to 25 g/L/h. At a reaction temperature of 45 °C, the catalyst specific activity was 692 U/g_CDW_ and a COS concentration of 38 g/L was obtained after just 1.5 h of reaction. The soluble COS yield (93%) was higher than at 30 °C (83%), reflecting better solubility of the COS at higher temperatures [60]. Moreover, the product composition slightly shifted towards longer-chain COS when the reaction was performed at 45 °C (Additional file 1: Fig. S5). This compositional shift occurred in consequence of a differential change in the specific activities of the individual enzymes, dependent on temperature. Upon a temperature increase from 30 °C to 45 °C, the CuCbP activity doubled (22.5 ± 1.4 U/mg; *N* = 6) while the CcCdP activity increased 3.3-fold (14.7 ± 1.6 U/mg; *N* = 5). Consequently, the oligomerization towards longer-chain COS was more pronounced at 45 °C than at 30 °C (Additional file 1: Fig. S5).

**Fig. 6:**
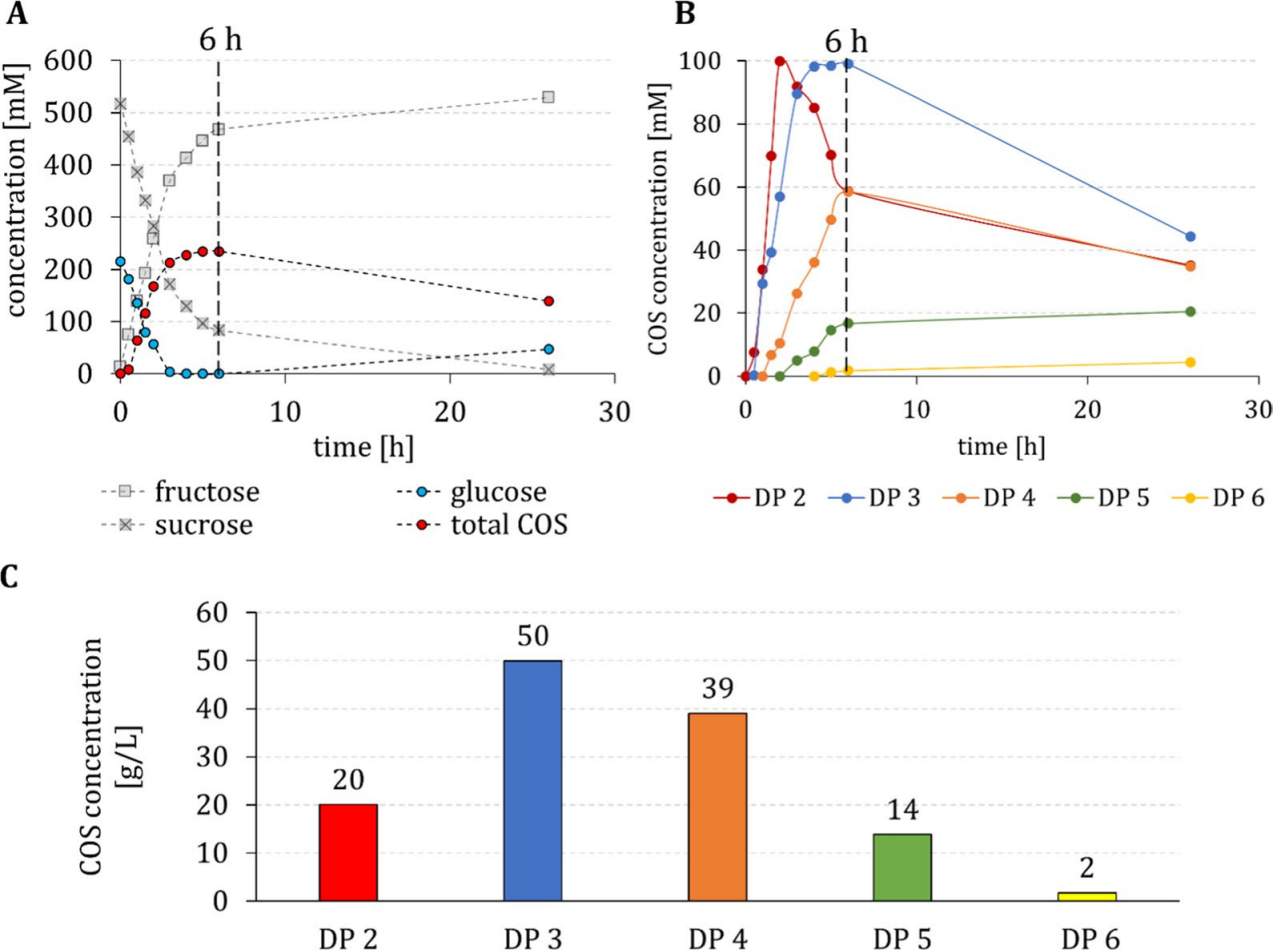
Optimized conversion catalysed by freeze–thaw permeabilized pPOLY_2 whole-cell catalyst at 45 °C. **A** Conversions of the substrates sucrose and glucose to fructose and total soluble COS. **B** Production of single soluble COS species over time (DP 2–6). **C** Product composition after 6 h reaction time

Zhong et al. found that the COS product solubility is controllable by the sucrose to glucose substrate ratio [2, 24]. Increased glucose concentrations reduce the elongation of longer-chain COS, since glucose functions as a primer for the COS synthesis. Thus, we decreased the sucrose:glucose ratio from 3.1 to 2.5 and, at the same time, increased the concentrations of both sugars from 200 mM:65 mM to 500 mM:200 mM. The reaction was performed using 3 g_CDW_/L freeze-thaw treated pPOLY_2 catalyst (Fig. 6). The activity ratio was 35:9.7:3.3 (each U/mL), as calculated from the specific activities of purified enzymes at 45 °C.

After 6 h of reaction, a COS concentration of 125 g/L (DP 2–6), a STY of 20 g/L/h and a total turnover number of 42 g_product_/g_CDW_ were obtained. Insoluble COS were only produced after 26 h and were not further analysed. The soluble COS yield on sucrose was ~ 84 mol % and on glucose 100 mol % after 6 h. We believe that these process parameters are highly promising for further development towards scale up and industrial implementation.

In comparison to the COS produced in this study (Fig. 6C), the product formed with the optimized bioconversion using isolated enzymes, published by Zhong et al. [2], contained a larger portion of longer-chain COS (DP 3: 33%; DP 4: 34%; DP 5: 24%; DP 6: 9%, all wt %). Possible reasons are the higher CcCdP activity (6 U/mL) compared to CuCbP (4 U/mL) as well as the lower glucose concentration (150 mM) [2]. Volumetric activities of the individual enzymes were lower than in the COS synthesis performed in this work, i.e., 20:6:4 vs. 35:9.7:3.3, U/mL. This could furthermore explain the slightly higher product concentration achieved with the whole-cell catalyst (105 g/L; DP 3–6) than with the purified enzymes (93 g/L; DP 3–6 [2]).

### COS production in context of commercial oligosaccharides

Enzymatic productions of established oligosaccharide prebiotics reach product concentrations of ~ 350 g/L fructo-oligosaccharides (FOS) and ~ 100 g/L galacto-oligosaccharides (GOS) [61]. The COS synthesis reported here is largely on par with these productions in terms of the reaction output. Previously, GOS synthesis was also carried out by using whole-cell catalysts [62–65]. The highest product concentration of 197 g/L GOS tri- and tetra-saccharides [65] was achieved with permeabilized *Lactococcus lactis* cells overexpressing the hyper-thermostable β-galactosidase from *Sulfolobus solfataricus*. Whole-cell syntheses of FOS [66–68] are generally performed with living cells (*Aureobasidium pullulans*) in repeated batch cultures. Under optimal fermentation conditions, a maximum FOS concentration of ~ 550 g/L with a productivity of ~ 10 g/L/h has been achieved just recently [68]. However, using growing cells in large-scale bioprocesses results in substantial amounts of biomass waste [69] and in most cases, cell growth involves release of carbon dioxide. Especially for productions in multiple cycles, a non-living cell factory, as shown in the current study, is considered best for a sustainable industrial bioprocess.

## Conclusions

Despite the increasingly sophisticated synthetic biology toolbox, fine-tuning of activity ratios is still challenging in cell-factory development. COS bottom-up production relies on three distinct phosphorylase activities and is based on the iterative elongation by CcCdP, which requires an even more precise activity control to avoid product loss by the formation of insoluble material. Yet, we were able to prevent production of insoluble COS by the co-expression of high amounts of recombinant protein (46% of total soluble *E. coli* protein) in a fine-tuned activity ratio of BaScP, CuCbP and CcCdP of 10:2.9:0.6. We achieved performance metrics, i.e., a product concentration of 125 g/L and a STY of 20 g/L/h for soluble COS production, which are comparable to production process data for other prebiotic oligosaccharides such as GOS [61]. Nevertheless, GOS production only requires one single enzyme and is therefore hardly comparable to the multi-enzyme cascade presented in this study. The more enzymes are involved in a bioconversion the more attractive the concept of a whole-cell catalyst becomes, as it saves costs, time, and waste. Future catalyst recycling and immobilization of the non-living cell catalyst designed here are considered promising and might enable the synthesis process to even surpass industry benchmarks in order to produce COS.

## Methods

### Plasmid construction

The construction of the pBICI co-expression plasmid harboring CuCbP (GenBank identifier AAQ20920.1) and BaScP (GenBank identifier AF543301.1) was described elsewhere [40]. Plasmids carrying CcCdP (GenBank identifier CDZ24361.1) were designed from in-house available DNA parts (Additional file 1: Table S1) using SnapGene® software (GSL Biotech LLC, Chicago, IL, United States). Synthetic DNA fragments and primers (Additional file 1: Table S5) were from Integrated DNA Technologies, Inc. (Carolville, IA, United States). Synthetic DNA fragments were assembled by overlap extension PCR [70] and restriction cloning. *E. coli* Top10F’ (Thermo Fisher Scientific Inc., Waltham, MA, United States) was used for plasmid amplification. Plasmids were sequence-verified by restriction analysis and sequencing (Microsynth Austria GmbH, Vienna, Austria). PCR products were isolated with GeneJET Gel Extraction Kit and plasmids were extracted using GeneJET Plasmid Miniprep Kit (both Thermo Fisher Scientific Inc.). Phusion Polymerase for PCR, restriction enzymes and T4 DNA ligase were from Thermo Fisher Scientific Inc.

### Cell preparation

*E. coli* BL21(DE3)*agp*^–^ (obtained from Tom Desmet, Ghent University, Belgium) was used for enzyme expression. Cells were transformed using electroporation [71] and regenerated in 1 ml SOC medium [72] for 1 h at 37 °C. Positive transformants were selected on lysogeny broth (LB)-agar plates containing 100 mg/L ampicillin.

For enzyme expression *E. coli* strains were grown at 37 °C in LB-medium (5 g/L NaCl, 5 g/L yeast extract, 10 g/L peptone from casein) in baffled shake flasks containing 100 mg/L ampicillin. For the cultivation of the two-plasmid catalysts, 100 mg/L ampicillin and 50 mg/mL kanamycin were added to the medium. To prepare the main culture, 250 mL medium in a 1 L flask were inoculated with cells from an overnight preculture to an optical density at 600 nm (OD_600_) of 0.03. Biomass was grown to an OD_600_ of 0.8–1.0 and expression was induced with 1 mM IPTG. Incubation occurred overnight at 25 °C and 110 rpm in incubation shaker CERTOMAT BS-1 (Sartorius, Göttingen, Germany). The OD_600_ was measured spectrophotometrically (DU® 800 UV/Vis Spectrophotometer, Beckman Coulter, Brea, CA, United States). Cell dry weight (CDW) was determined by filtering 15–20 mL cell culture through a pre-weighed Whatman® Nuclepore™ TrackEtched membrane (diameter 50 mm, pore size 0.4 µm, polycarbonate, Sigma Aldrich/Merck, Darmstadt, Germany). The cells were washed with 10–15 mL water, were dried overnight at 70 °C and weighed. Centrifuged cells (20 min, 4 °C, 4.4 krcf, Ultracentrifuge Sorvall RC-5B Superspeed, Thermo Fisher Scientific Inc.) were resuspended in 50 mM MES buffer, pH 7.0 (6:1 cell wet weight, v:w), aliquoted (~ 10–15 mL portions) and stored at − 70 °C until further use.

To prepare the cell lysate, an aliquot of the cell suspension was thawed and ultra-sonicated (Branson Ultrasonics™ Microtips Probe 1/8′′ dia 418-A, Thermo Fisher Scientific Inc.) using 3 times a 6-min run time (2 s pulse on, 4 s pulse off, 30% amplitude). Centrifuged cell extract (21.1 krcf, 4 °C for 45 min, Centrifuge Eppendorf 5424 R, Eppendorf, Hamburg, Germany) was immediately used for enzyme activity determination and subsequently stored at − 20 °C.

Enzymes were purified via their N-terminal hexahistidine-tag or Strep-tag II. Purified proteins were desalted using the Vivaspin Turbo 10 kDa (BaScP) or 30 kDa (CuCbP and CcCdP) cutoff concentrator tubes (Sartorius Stedim, Vienna, Austria) with MES buffer (50 mM, pH 7.0).

### Expression analysis and enzyme activity assays

BaScP activities from both, isolated and mixed enzyme preparations (cell-free extract from co-expression) were determined in the phosphorolysis direction by a continuous coupled activity assay [73]. The αGlc1-*P* liberated upon enzyme action (synthesized from 250 mM sucrose to 50 mM phosphate) was converted by phosphoglucomutase from rabbit muscle (3 U/mL, Sigma-Aldrich/Merck, Darmstadt, Germany) and NAD^+^-dependent d-glucose-6-phosphate dehydrogenase from *Leuconostoc mesenteroides* (3.4 U/mL, Sigma-Aldrich/Merck) to NADH, which then was monitored spectrophotometrically at 340 nm (DU® 800 UV/Vis Spectrophotometer Beckman Coulter). Measurements were performed at 30 °C. BaScP activities at 45 °C were determined discontinuously: αGlc1-*P* release from 250 mM sucrose to 50 mM phosphate (50 mM MES buffer, pH 7.0) was equally converted to NADH and measured spectrophotometrically at 340 nm. One unit (U) of activity is the enzyme amount producing 1 μmol αGlc1-*P*/min under the conditions employed. Further details can be found elsewhere [40].

CuCbP phosphorolysis activities at 30 °C (isolated and co-expressed enzyme preparations) were determined continuously, similar to BaScP activity measurements. Instead of sucrose, 50 mM cellobiose was used to start the reaction. Glucose was not present in either assay condition, which allowed activity measurements of BaScP and CuCbP without affecting the activity of the other. CuCbP phosphorolysis activity was additionally measured at 45 °C discontinuously, as described above, with 50 mM cellobiose instead of 250 mM sucrose. The synthesis activity of CuCbP could only be measured from the purified enzyme, as the present CcCdP activity would interfere with the measurement. Therefore, the synthesis activities stated in the "Results and discussion" section (Fig. 3) were calculated based on the synthesis/phosphorolyses ratio measured from the purified enzyme. 50 mM glucose and 50 mM αGlc1-*P* (in 50 mM MES buffer, pH 7.0) were converted to cellobiose. Phosphate release from αGlc1-*P* was measured using the colorimetric assay of Saheki et al. [74]. Reactions were performed at 30 °C and 45 °C. One unit (U) of activity is the enzyme amount producing 1 μmol phosphate/min under the conditions employed.

CcCdP activity was determined in cellodextrin synthesis direction (50 mM *p*-nitrophenyl β-D-cellobioside (*p*NP-G2), 50 mM αGlc1-*P*, pH 7.0, 30 °C or 45 °C in 50 mM MES buffer, pH 7.0) by the colorimetric assay of Saheki et al. [74]. *p*NP-G2 (CarboSynth, Compton, Berkshire, United Kingdom) was used as the acceptor substrate to avoid possible interference from the simultaneously present CuCbP activity in cell-free extracts [28]. If cellobiose had been used, as is done in the regular assay for CcCdP activity [23], the CuCbP could have used the cellobiose as substrate for phosphorolysis once sufficient phosphate had become released by the activity of the CcCdP. Activities of the purified CcCdP were compared on cellobiose and *p*NP-G2, each at 30 °C and 45 °C (Additional file 1: Table S6). Measurements at 45 °C for both glycosyl-acceptors were confirmed by literature values [25]. Stated activities in the "Results and discussion" section (Fig. 3) were normalized for cellobiose as the acceptor substrate.

Protein concentration was determined according to the manual of Carl Roth GmbH (Karlsruhe, Germany). 10 μL sample was mixed with 500 μL of the 1 × Roti®-Qant dye (Carl Roth GmbH). After 15 min incubation at room temperature, absorbance was measured at 595 nm (DU® 800 UV/Vis Spectrophotometer Beckman Coulter). The protein concentration was calculated from a calibration curve in the range of 0.1 to 1.0 g/L bovine serum albumin (Sigma-Aldrich/Merck). Expression of the enzymes was analyzed by SDS-PAGE (Additional file 1: Page 11). The portion of enzyme in total soluble *E. coli* protein was calculated from specific activities in cell-free extracts and isolated enzymes.

### Cellodextrin synthesis in whole-cell and cell-free systems

The reaction mixture consisted of either 200 mM:65 mM sucrose:glucose or 500 mM:200 mM sucrose:glucose, 50 mM phosphate, 50 mM MES buffer (pH 7.0) and whole-cell catalyst (2.6 g_CDW_/L or 3 g_CDW_/L) or a corresponding volume of cell-free extract. Reactions in 50 mL total volume were performed in 100 mL borosilicate glass bottles equipped with Rotilabo® magnetic sticks (25 × 8 mm, Carl Roth GmbH, Karlsruhe, Germany) at 30 °C or 45 °C, pH 7.0 and 300 rpm for 26 h on a Variomag® Multi-Magnetic Stirrer (Thermo Fisher Scientific Inc.).

Activities of thawed cell-free extracts were measured shortly prior to the reaction start. Cell-free extracts and thawed cell suspensions (semi-permeabilized cells) were treated similarly. It was assumed that semi-permeabilized cells show similar activities as the corresponding cell-free extracts (prepared from the same cultivation batch). A master mix containing all reaction components was distributed to the bottles. Reactions were started by adding the catalysts.

Samples were periodically collected, heated at 99 °C for 10 min to inactivate the enzymes (ThermoMixer C, E-5048, Eppendorf), and centrifuged for 10 min at 21.1 krcf (Centrifuge Eppendorf 5424 R, Eppendorf). The supernatant was stored at − 20 °C until HPLC measurements were performed. αGlc1-*P* concentration of some samples was determined spectrophotometrically following the discontinuous assay in "Expression analysis and enzyme activity assays" section.

### HPLC analysis of reaction compounds

Quantification of main components in the reaction mixtures was performed by HPLC using a Merck Hitachi L-7100 system (Merck, Darmstadt, Germany) equipped with an autosampler (L-7250) and a refractive index detector (L-7490). Separation of sucrose, glucose, fructose and cellobiose was performed with an YMC-Pack Polyamine II/S-5 µm/12 nm column (250 mm × 4.6 mm; YMC Co., Ltd., Shimogyo-ku, Kyoto, Japan). Additionally, a guard column (20 mm × 4.0 mm; YMC Co., Ltd.) was installed. Elution was performed isocratically with an acetonitrile–water mixture (75:25, v:v) at a flow rate of 1 mL/min. Measurements were performed at room temperature, the injection volume per sample was set to 20 µL and the running time was 35 min. Soluble COS (DP 2–6) were quantified by a Luna 5 µm NH2 column (100 Å, 250 × 4.6 mm, Phenomenex, Aschaffenburg, Germany) operated at 40 °C. Acetonitrile–water (70:30, v:v) was used as eluent at a flow rate of 1.5 mL/min and a running time of 15 min.

Representative chromatograms of product solutions measured by the stated HPLC methods are shown in Additional file 1: Figure S6. Refractive index detected peaks were analyzed using the software Chromeleon Chromatography Data System (Thermo Fischer Scientific Inc.). Calibration was done with standards containing main components. Reagent-grade COS standards ranging from DP 2–6 were from CarboSynth (Compton).

Insoluble COS production was calculated based on the molar balance between the sum of glucose units used as primers for individual soluble COS species and the actually consumed glucose units by subtracting moles of soluble COS (DP 2–6) from moles converted glucose.

## Supplementary Information

**Additional file 1:** **Table S1**. Parts used for the construction of the co-expression plasmids. **Table S2.** Enzymatic synthesis activities corresponding to 2.6 g_CDW_/L reaction mixtures used in whole-cell COS productions with single-plasmid catalysts (Figure S4). Activities were measured in corresponding cell-free extracts (see the "Methods" section in "Expression analysis and enzyme activity assays" sub section). **Table S3.** COS synthesis with different pPOLY_2 whole-cell catalyst preparations. **Table S4**. Enzyme activities measured in the supernatant of the different pPOLY_2 catalyst preparations; cell suspensions had a concentration of 25 mg_CDW_/mL. A cell-free extract preparation was measured as the reference for 100% activity. **Table S5**. Primers used to construct the co-expression plasmids containing CcCdP. **Table S6. **Specific activities of purified CcCdP on cellobiose and *p*NP-G2 at two temperatures. **Figure S1. **SDS PAGE gel (top) shows soluble protein of co-expression catalysts. Two-plasmid approach (p15A + pBICI med *ori*) strongly expressed CcCdP but not CuCbP nor BaScP. After purification and restriction analysis of both plasmids, agarose gel electrophoresis (bottom) revealed a reduced size of pBICI med *ori* (~3000 bp instead of 7379 bp). Sample **1** is the overnight preculture (not induced), samples **2** and **3** are duplicates of induced expression cultures. **Figure S2.** SDS-PAGE analysis of expression optimization. (**A**) Tested parameters were expression durations (after 4 hours and 18 hours) and IPTG concentrations (0.1 mM and 1 mM) at 25 °C expression temperature. (**B**) pPOLY_2 expression was further analysed at two different temperatures (a, b) and four different IPTG concentrations (1-4). The cyan arrow marks 6xHis-CcCdP (112.8 kDa); dark green arrow, 6xHis-CuCbP (92.7 kDa); light green arrow, Strep II-BaScP (57.7 kDa). *NI*, non-induced sample. **Figure S3.** Plasmid maps of one-plasmid approaches (A-C) and two-plasmid approaches (D-F). The plasmid maps and full sequences are stored in the add gene database, accessible with the numbers 179272 to 179276 (https://www.addgene.org). **Figure S4.** Conversions of one-plasmid cell catalysts: (**A**) pDUBI, (**B**) pPOLY_1, (**C**) pPOLY_2. **Figure S5.** Temperature dependence of the COS composition. Comparison of COS species (DP 2-6) after 8 hours reaction at 30 °C (first bar; see also Figure 4, pPOLY_2) and after 1.5 h and 2 h reaction at 45 °C (second and third bar). Reactions were performed with freeze-thaw treated whole cell catalysts carrying the plasmid pPOLY_2. **Figure S6.** HPLC chromatograms of product solutions. Mono- and disaccharides were measured with an YMC-Pack Polyamine II/S-5 µm/12 nm column (A) and oligosaccharides (cellodextrins DP 3 to DP 6) were measured with a Luna 5 µm NH2 column (B).

## Data Availability

The datasets supporting the conclusions of this article are available in the zenodo repository, https://doi.org/10.5281/zenodo.5148144.

## Abbreviations

αGlc1-*P*: α-D-glucose-1-phosphate
BaScP: Sucrose phosphorylase from *Bifidobacteium adolescentis*
CDW: Cell dry weight
CcCdP: Cellodextrin phosphorylase from *Clostridium* *cellulosi*
COS: Cello-oligosaccharides
CuCbP: Cellobiose phosphorylase from *Cellulomonas uda*
DP: Degree of polymerization
FOS: Fructo-oligosaccharides
GOS: Galacto-oligosaccharides
IPTG: Isopropyl β-D-1-thiogalactopyranoside
MES: 2-(*N*-Morpholino)ethanesulfonic acid
pBICI: Bicistronic co-expression plasmid
pDUBI: Mono- and bicistronic co-expression plasmid
*p*NP-G2: *p*-Nitrophenyl β-D-cellobioside
pPOLY: Tricistronic co-expression plasmid
RBS: Ribosome binding site
STY: Space–time yield
TTN: Total turnover number
