# Supplementary material for: Engineering cascade biocatalysis in whole cells for bottom-up synthesis of cello-oligosaccharides: flux control over three enzymatic steps enables soluble production
Source: Microb Cell Fact. 2022 Apr 9;21:61. doi: 10.1186/s12934-022-01781-w (PMC8994397; doi:10.1186/s12934-022-01781-w)
Supplement: Supplementary file 1 — Additional file 1: Table S1. Parts used for the construction of the co-expression plasmids. Table S2. Enzymatic synthesis activities corresponding to 2.6 gCDW/L reaction mixtures used in whole-cell COS productions with single-plasmid catalysts (Figure S4). Activities were measured in corresponding cell-free extracts (see the "Methods" section in "Expression analysis and enzyme activity assays" sub section). Table S3. COS synthesis with different pPOLY_2 whole-cell catalyst preparations. Table S4. Enzyme activities measured in the supernatant of the different pPOLY_2 catalyst preparations; cell suspensions had a concentration of 25 mgCDW/mL. A cell-free extract preparation was measured as the reference for 100% activity. Table S5. Primers used to construct the co-expression plasmids containing CcCdP. Table S6. Specific activities of purified CcCdP on cellobiose and pNP-G2 at two temperatures. Figure S1. SDS PAGE gel (top) shows soluble protein of co-expression catalysts. Two-plasmid approach (p15A + pBICI med ori) strongly expressed CcCdP but not CuCbP nor BaScP. After purification and restriction analysis of both plasmids, agarose gel electrophoresis (bottom) revealed a reduced size of pBICI med ori (~3000 bp instead of 7379 bp). Sample 1 is the overnight preculture (not induced), samples 2 and 3 are duplicates of induced expression cultures. Figure S2. SDS-PAGE analysis of expression optimization. (A) Tested parameters were expression durations (after 4 hours and 18 hours) and IPTG concentrations (0.1 mM and 1 mM) at 25 °C expression temperature. (B) pPOLY_2 expression was further analysed at two different temperatures (a, b) and four different IPTG concentrations (1-4). The cyan arrow marks 6xHis-CcCdP (112.8 kDa); dark green arrow, 6xHis-CuCbP (92.7 kDa); light green arrow, Strep II-BaScP (57.7 kDa). NI, non-induced sample. Figure S3. Plasmid maps of one-plasmid approaches (A-C) and two-plasmid approaches (D-F). The plasmid maps and full sequences are stored in t [file 12934_2022_1781_MOESM1_ESM.docx]

*Supplementary Information*

**Engineering cascade biocatalysis in whole cells for bottom-up synthesis of cello-oligosaccharides: flux control over three enzymatic steps enables soluble production**

Katharina N. Schwaiger^1^, Alena Voit^1^, Birgit Wiltschi^1^, Bernd Nidetzky^1,2^

^1^acib - Austrian Centre of Industrial Biotechnology, Graz, Austria

^2^Institute of Biotechnology and Biochemical Engineering, Graz University of Technology, NAWI Graz, Graz, Austria

**Correspondence:** Bernd Nidetzky, Institute of Biotechnology and Biochemical Engineering, TU Graz, NAWI Graz, Petersgasse 12, 8010 Graz, Austria

**E-mail**: [bernd.nidetzky@tugraz.at](mailto:bernd.nidetzky@tugraz.at)

**Keywords:** Cello-oligosaccharides, multi-enzymatic cascade, whole-cell catalyst, co-expression, activity ratios

**Content**

Table S1, S2.………………………………………………………………………………………………………………………..…2

Table S3, S4………………………………..……………………………………………………………………………………….....3

Table S5, S6………………………………………………………………….………………………………………………….….…4

Figure S1…….……………….…………………………………………………………………..………………………………..…...5

Figure S2………...………………………………………………………………………………………………………………….….6

Figure S3………………………………………………………………………………………………………………………………..7

Figure S4………………………………………………………………………………………………………………………………..8

Figure S5……………..…..……………………………………………………………………………………………………………..9

Figure S6………………………………………………………………………………………………………………………………10

*Methods*: Details to SDS PAGE………………………………………………………………………………………………..11

References...………………………………………………………………………………………………………………………….12

**Table S1**. Parts used for the construction of the co-expression plasmids.

| Part | | Description | Reference |
| --- | --- | --- | --- |
| Origin of replication | pUC | high copy number origin of replication (500-700 copies per cell [1])  template: pUC19c | [2] |
|  | pBR322 | medium-copy number origin of replication (15-20 copies per cell [1])  template: pQE30 | [3] |
|  | p15A | medium-copy number origin of replication (18-22 copies per cell [1]) | [2] |
| Selection marker | Amp^R^ | ampicillin resistance | [4] |
|  | Kan^R^ | kanamycin resistance | [5] |
| Promoter | P_T7_*_lacO_* | strong promoter from bacteriophage T7  regulated by *lacO* site  for use with *E. coli* BL21(DE3)  inducible with IPTG | [6] |
|  | P_T5_*_lacO_* | strong promoter from bacteriophage T5  regulated by 2 *lacO* sites  inducible with IPTG | [7,8]^,^[9,10] |
| RBSs | weak | UCACACAGGACacccccAUG | [http://parts.igem.org/Part: BBa_B0033] |
|  | medium | UcacacaggaaagacccccAUG | [http://parts.igem.org/Part: BBa_B0032] |
|  | strong | AaagaggagaaaacccccAUG | [11] |
|  | T7 | efficient RBS from bacteriophage T7 gene 10 | [12] |
| Regulators | LacI | originates from *E. coli* strain K-12 substrain MG1655 | [13] |
| Terminators | *rrnB*T1 | originate from *E. coli* strain K-12 substrain MG1655 | [13] |
|  | *rrnB*T2 |  |  |
|  | *rrnC* |  |  |
|  | λt0 | originates from bacteriophage λt0 | [14] |
|  | T7 | originates from bacteriophage T7 | [15] |

RBS, ribosome binding site

| **Cell catalyst** | **BaScP (U/mL)** | **CuCbP (U/mL)** | **CcCdP (U/mL)** |
| --- | --- | --- | --- |
| **pPOLY_2** | 15.2 | 4.2 | 0.9 |
| **pPOLY_1** | 18.8 | 1.6 | 1.8 |
| **pDUBI** | 10.0 | 2.8 | 0.5 |

**Table S2:** Enzymatic synthesis activities corresponding to 2.6 g_CDW_/L reaction mixtures used in whole-cell COS productions with single-plasmid catalysts (Figure S4). Activities were measured in corresponding cell-free extracts (see the Methods section Expression Analysis).

**Table S3:** COS synthesis with different pPOLY_2 whole-cell catalyst preparations.

|  | **soluble COS yield (8 h)** | **initial cell catalyst activity (1h)** | **product titer**  **(8h)** | **STY**  **(8h)** | **TTN**  **(8h)** |
| --- | --- | --- | --- | --- | --- |
|  | *%* | *U/g_CDW_* | *g/L* | *g/L/h* | *g_Product_/g_CDW_* |
| **freeze-thaw treated cells** | 83% | 315 | 27 | 3.4 | 10.3 |
| **cell-free extract** | 88% | 384 | 37 | 4.6 | 14.0 |
| **freeze-dried cells** | 85% | 281 | 28 | 3.5 | 10.6 |
| **non-permeabilized cells** | 11% | 5.0 | 2.5 | 0.3 | 1.0 |

**Table S4**: Enzyme activities measured in the supernatant of the different pPOLY_2 catalyst preparations; cell suspensions had a concentration of 25 mg_CDW_/mL. A cell-free extract preparation was measured as the reference for 100% activity.

|  | ***Enzmye*** | ***activity (U/mL)*** | ***leakiness (%)*** |
| --- | --- | --- | --- |
| **cell-free extract** | *BaScP* | 153.1 ± 2.1 | 100% |
|  | *CuCbP* | 43.0 ± 1.4 | 100% |
|  | *CcCdP* | 9.3 ± 0.9 | 100% |
| **freeze-thaw treated cells** | *BaScP* | 2.82 ± 0.12 | 1.8% |
|  | *CuCbP* | 0.58 ± 0.02 | 1.3% |
|  | *CcCdP* | 0.13 ± 0.01 | 1.4% |
|  | ***total*** |  | **4.5%** |
| **non-permeabilized cells** | *BaScP* | 1.18 ± 0.04 | 0.8% |
|  | *CuCbP* | 0.26 ± 0.01 | 0.6% |
|  | *CcCdP* | 0.04 ± 0.01 | 0.4% |
|  | ***total*** |  | **1.8%** |
| **freeze-dried cells** | *BaScP* | 7.20 ± 0.50 | 4.7% |
|  | *CuCbP* | 1.44 ± 0.12 | 3.3% |
|  | *CcCdP* | 0.35 ± 0.06 | 3.8% |
|  | ***total*** |  | **12%** |

**Table S5**. Primers used to construct the co-expression plasmids containing CcCdP

|  | # | Sequence 5’-3’ |
| --- | --- | --- |
| sequencing CcCDP | pBP2340 | ATGACAACATCCTGGAAGCG |
|  | pBP2341 | GGGTGTCAACCATAATGCCG |
| pPOLY construction | pBP2565 | ccctctagaaataattttgtttaactttaagaaggagaACTAGTATGAGAGGATCGCATCACCATCACCATCACGgctccATTACCAAAG |
|  | pBP2566 | CGaGTCGACccgtgatggtgatggtgatggctgctgcCCATGCTAGCtttctcctcttTGCGGCCGCTTACTTGAACTCCAGGGTCACGCTATGCTC |
|  | pBP2614 | gTAGTGTACAAaagaggagaaaACTAGTATGAGAGGATCGCATCACCATCACCATCACgg |
|  | pBP2615 | tcttTTGTACACAATTGTTACTTGAACTCCAGGGTCACGCTATGCTC |
| sequencing CcCDP | pBP2616 | GCCGGTGTAGGTGATGCTCAGG |
|  | pBP2617 | AGCAGCGCGCTGAGCG |
| p15A-CcCDP construction | pBP2618 | ataCTCGAGtaaaaaaaatccttagctttcgctaaggatGTGC |
|  | pBP2619 | actggaaagcgggcagtgaAGATCTATTAAAGAGGAGAAAGTCGACATGATTGAACAAGATGGATTGCACGCAGGTTC |
|  | pBP2620 | GAATTCgacaccatcgaatggtgcaaaacc |
|  | pBP2621 | ggttttgcaccattcgatggtgtcGAATTCCTAGCGGAGTGTATACTGGCTTACTATG |
|  | pBP2622 | CCGCTCACAATTCCCCTATAGTGAGTCGTATTAACTGCAGacaacttatatcgtatggggctgacttcAGGTGC |
|  | pBP2623 | ttaCTCGAGtattatAAGCTTCAAAAAACCCCTCAAGACCCGTTTAGAGGC |
| pDUBI construction | pBP2497 | ataGGATCCAAATCATAAAAAATTTATTTGCTTTGTGAGCG |
|  | pBP2714 | CTCTCATACTAGTtttctcctctttagattcTGTGTGAAATTGTTATCCGCTCACAATTG |
|  | pBP2715 | CACACAgaatctaaagaggagaaaACTAGTATGAGAGGATCGCATCACCATC |
|  | pBP2716 | TTAGGATCCTAAAAAAAATCCTTAGCTTTCGCTAAGGATGCTAGCTTACTTGAACTCCAGGGTCACGCTATGC |

**Table S6.** Specific activities of purified CcCdP on cellobiose and *p*NP-G2 at two temperatures

| temp | *p*NP-G2 [U/mg] | cellobiose [U/mg] | Relative activity [%]^1^ |
| --- | --- | --- | --- |
| 30 °C | 8.4 ± 0.8 | 4.4 ± 0.5 | **191** |
| 45 °C | 17.3 ± 1.7 | 14.7 ± 1.6 | **118** |

^1^ Relative activity [%] of *p*NP-G2 based on cellobiose [100%]


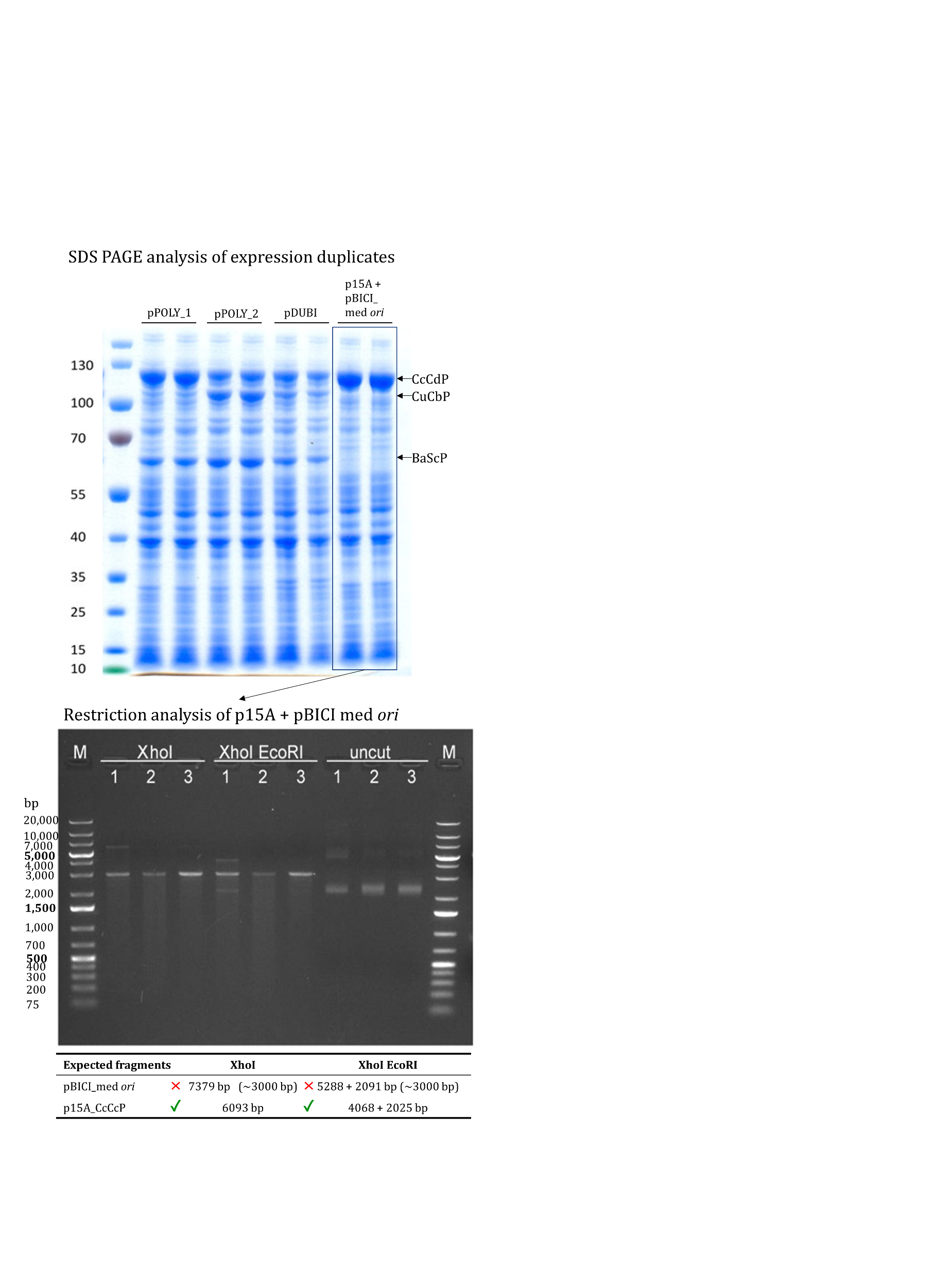


**Figure S1:** SDS PAGE gel (top) shows soluble protein of co-expression catalysts. Two-plasmid approach (p15A + pBICI med *ori*) strongly expressed CcCdP but not CuCbP nor BaScP. After purification and restriction analysis of both plasmids, agarose gel electrophoresis (bottom) revealed a reduced size of pBICI med *ori* (~3000 bp instead of 7379 bp). Sample **1** is the overnight preculture (not induced), samples **2** and **3** are duplicates of induced expression cultures.


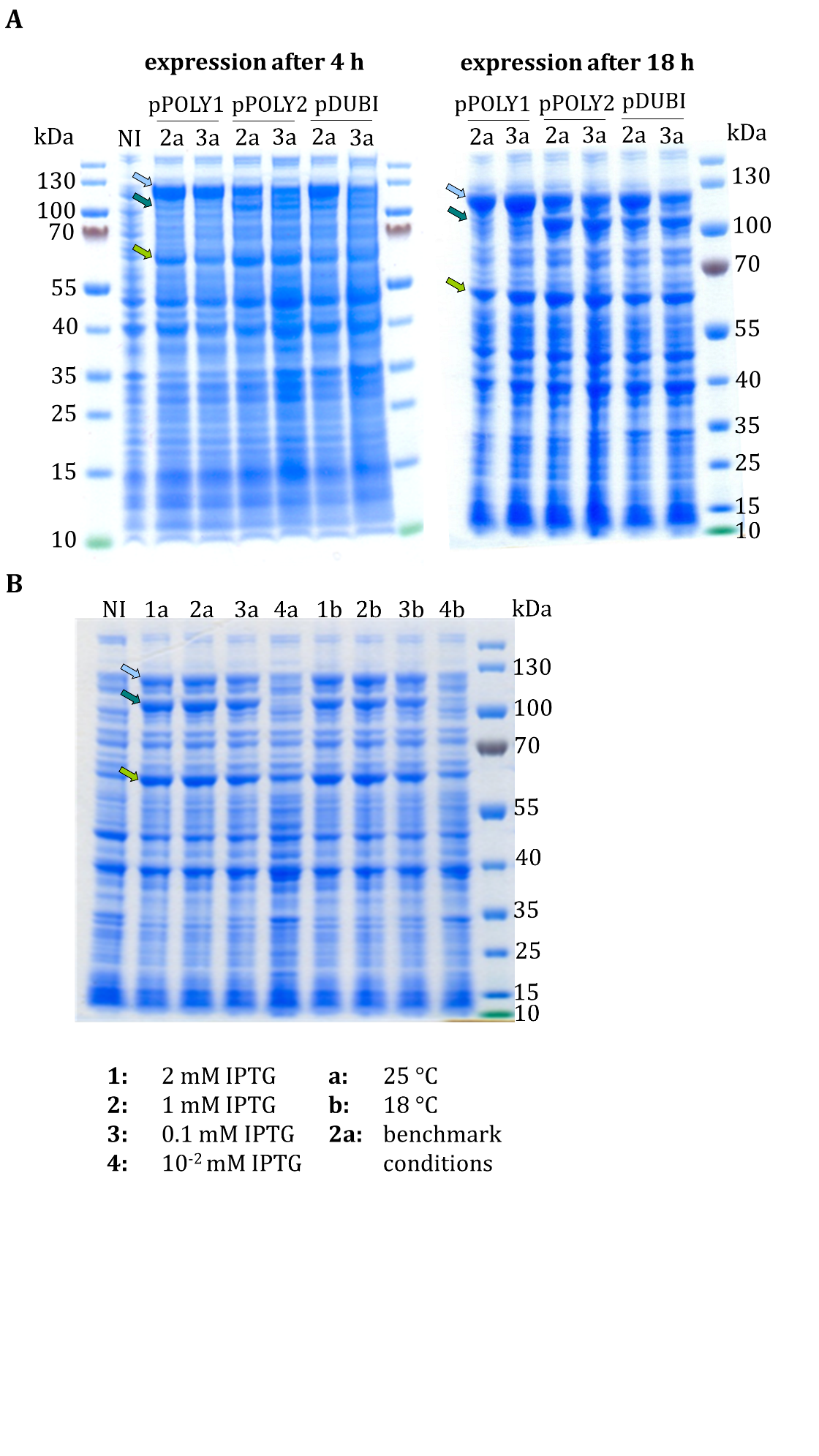


**Figure S2**: SDS-PAGE analysis of expression optimization. (**A**) Tested parameters were expression durations (after 4 hours and 18 hours) and IPTG concentrations (0.1 mM and 1 mM) at 25 °C expression temperature. (**B**) pPOLY_2 expression was further analysed at two different temperatures (a, b) and four different IPTG concentrations (1-4). The cyan arrow marks 6xHis-CcCdP (112.8 kDa); dark green arrow, 6xHis-CuCbP (92.7 kDa); light green arrow, Strep II-BaScP (57.7 kDa). *NI*, non-induced sample.


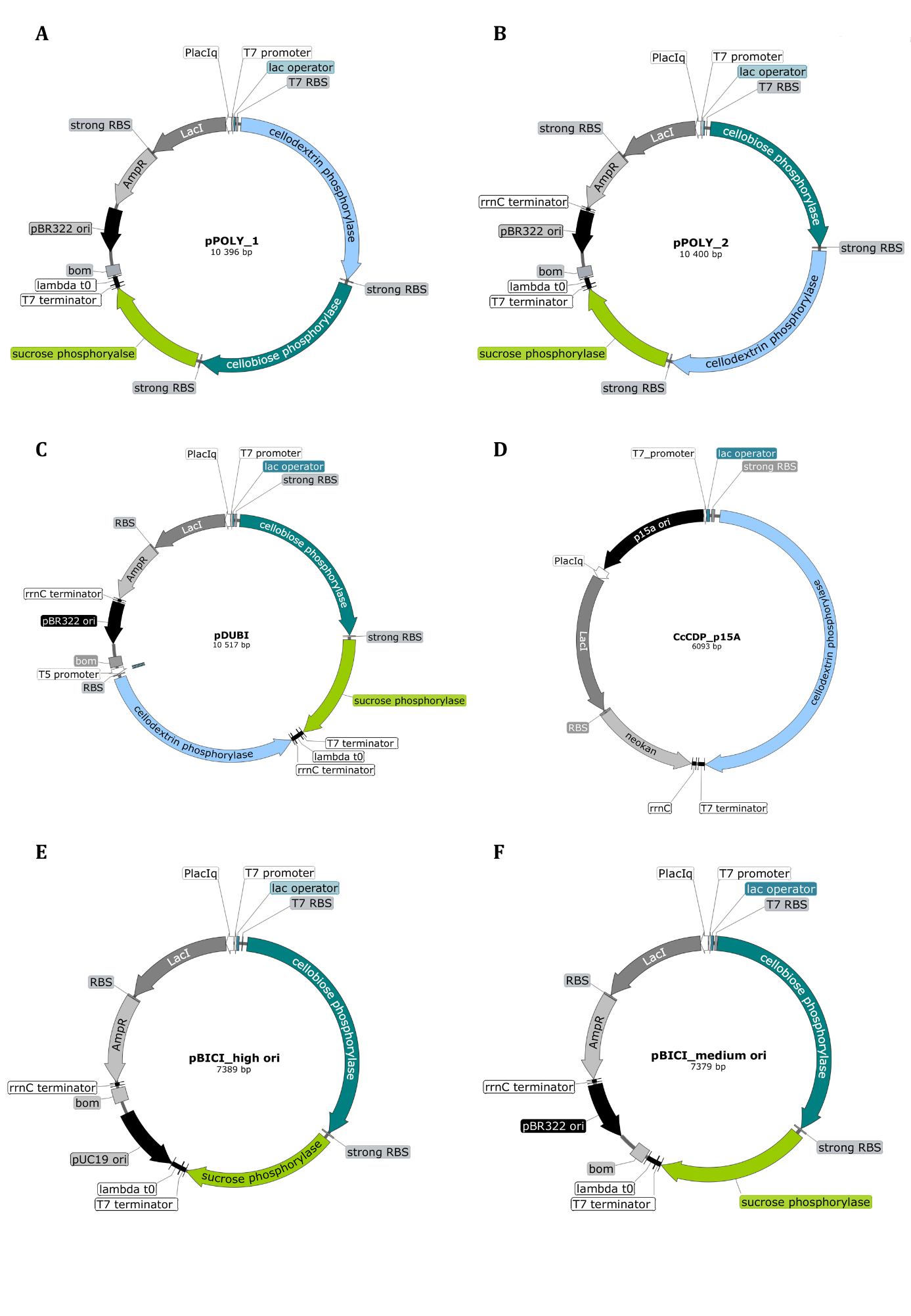


**Figure S3**: Plasmid maps of one-plasmid approaches (A-C) and two-plasmid approaches (D-F). The plasmid maps and full sequences are stored in the add gene database, accessible with the numbers 179272 to 179276 (https://www.addgene.org).

**
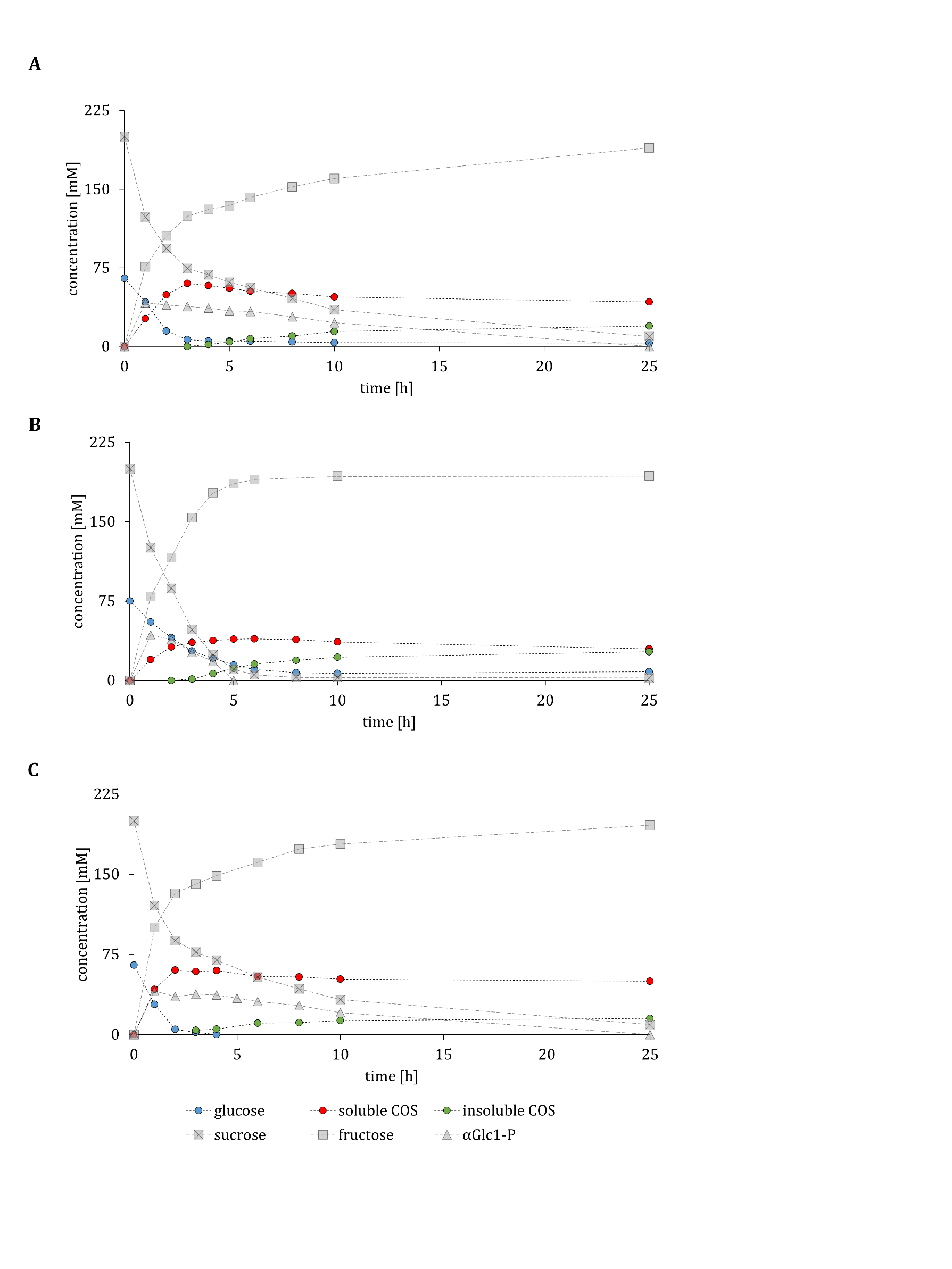
**

**Figure S4**: Conversions of one-plasmid cell catalysts: (**A**) pDUBI, (**B**) pPOLY_1, (**C**) pPOLY_2


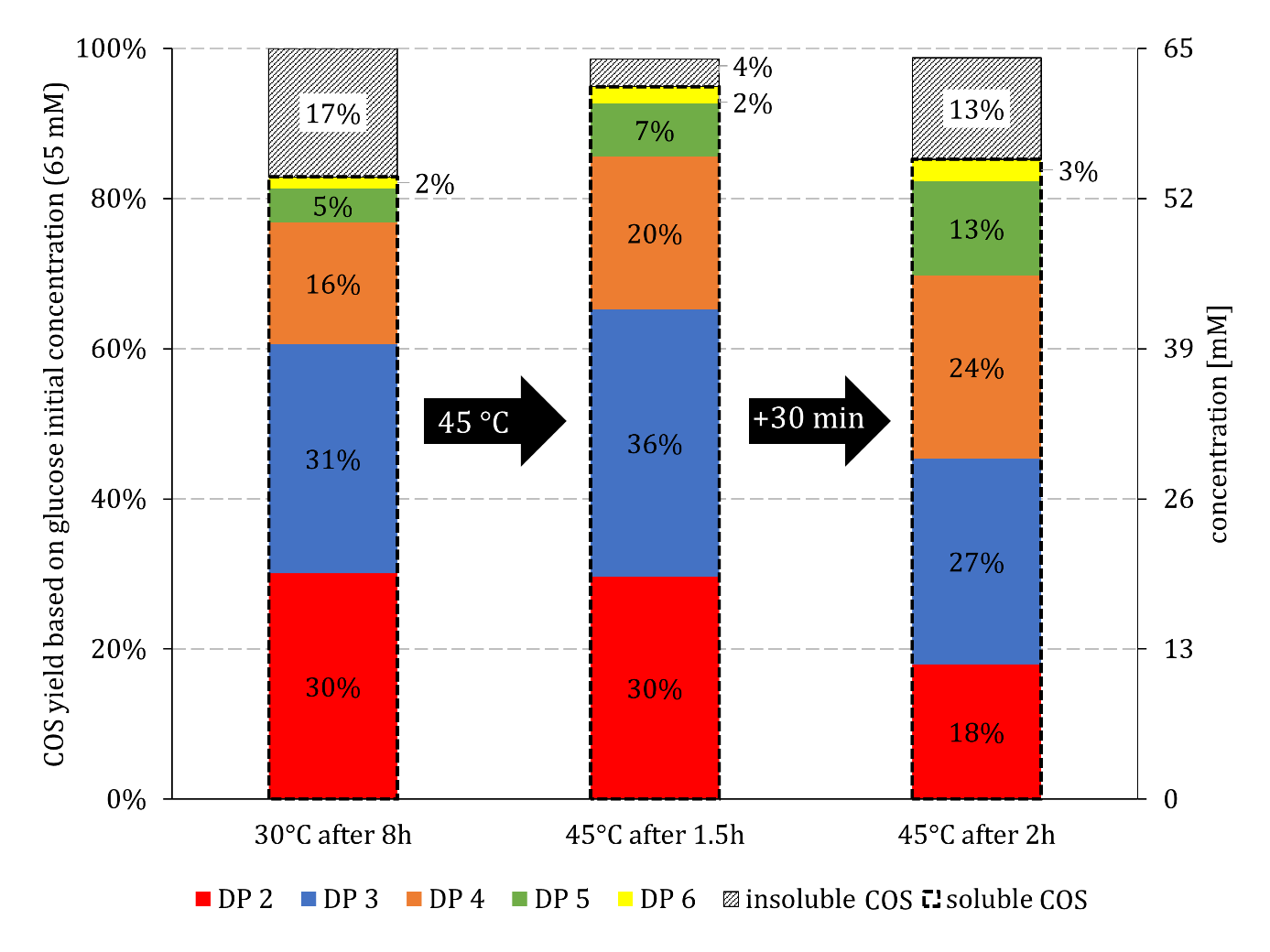


**Figure S5:** Temperature dependence of the COS composition. Comparison of COS species (DP 2-6) after 8 hours reaction at 30 °C (first bar; see also Figure 4, pPOLY_2) and after 1.5 hours and 2 hours reaction at 45 °C (second and third bar). Reactions were performed with freeze-thaw treated whole cell catalysts carrying the plasmid pPOLY_2.


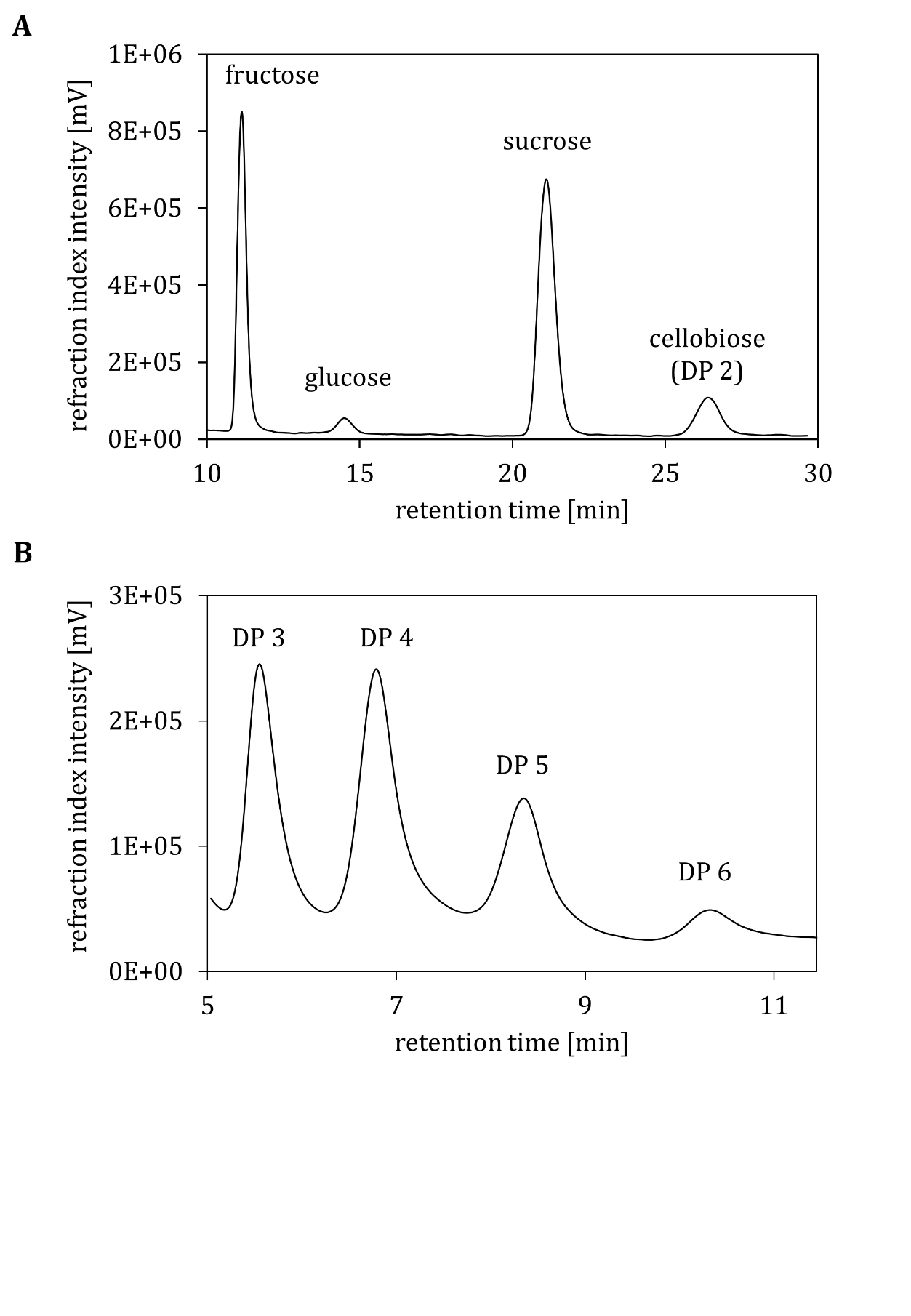


**Figure S6**: HPLC chromatograms of product solutions. Mono- and disaccharides were measured with an YMC-Pack Polyamine II/S-5 µm/12 nm column (A) and oligosaccharides (cellodextrins DP 3 to DP 6) were measured with a Luna 5 µm NH2 column (B).

# Methods

Expression analysis

Details to SDS-PAGE

10 μL of the properly diluted soluble and insoluble protein fractions were mixed with 4 μL water, 5 μL 4xLDS buffer (NuPAGE, Thermo Fisher Scientific Inc., Waltham, MA, United States) and 1 μL 1 M DTT, heated to 99 °C for 10 min and loaded on a NuPAGE 4-12% Bis-Tris Protein Gel (Thermo Fisher Scientific Inc.). The protein separation was performed at 175 V for 75 min in 1xMOPS buffer (NuPAGE MOPS SDS Running Buffer 20x, Thermo Fisher Scientific Inc.) in an XCell SureLock SDS gel chamber (Thermo Fisher Scientific Inc.). After completion of the run, the gel was stained with staining solution (75:500:425; acetic acid:ethanol:water; v:v:v; 2.5 g L-1 of Brilliant blue R250) for 30 min and destained (75:200:725; acetic acid:ethanol:water, v:v:v) to visualize the protein bands.

**References**

[1] Baek C-H, Liss M, Clancy K, Chesnut J, Katzen F. DNA Assembly Tools and Strategies for the Generation of Plasmids. Microbiol Spectr. 2014;2:1–12.

[2] Chang ACY, Cohen SN. Construction and characterization of amplifiable DNA cloning vectors derived from P15A cryptic plasmid. J Bacteriol. 1978;134:1141–56.

[3] Bolivar F, Rodriguez RL, Greene PJ, Betlach MC, Heyneker HL, Boyer HW, et al. Construction and characterization of new cloning vehicles. II. A multipurpose cloning system. Gene. 1977;2:95–113.

[4] Sutcliffe JG. Nucleotide sequence of the ampicillin resistance gene of *Escherichia coli* plasmid pBR322. Proc Natl Acad Sci. 1978;75:3737–41.

[5] Beck E, Ludwig G, Auerswald E., Reiss B, Schaller H. Nucleotide sequence and exact localization of the neomycin phosphotransferase gene from transposon Tn5 . Gene. 1982;19:327–36.

[6] Dubendorfft JW, Studier W. Controlling Basal Expression in an Inducible T7 Expression System by Blocking the Target T7 Promoter with *lac* Repressor. J Mol Biol. 1991;219:45–59.

[7] Brunner M, Bujard H. Promoter recognition and promoter strength in the *Escherichia coli* system. EMBO J. 1987;6:3139–44.

[8] Lanzer M, Bujard H. Promoters largely determine the efficiency of repressor action. Proc Natl Acad Sci. 1988;85:8973–7.

[9] Sadler JR, Sasmor H, Betz JL. A perfectly symmetric *lac* operator binds the *lac* repressor very tightly. Proc Natl Acad Sci. 1983;80:6785-9.

[10] Oehler S, Amouyal M, Kolkhof P, von Wilcken-Bergmann B, Müller-Hill B. Quality and position of the three *lac* operators of *E. coli* define efficiency of repression. EMBO J. 1994;13:3348–55.

[11] Elowitz MB, Leibler S. Nature. 2000;403:335–8.

[12] Olins PO, Rangwala SH. A novel sequence element derived from bacteriophage T7 mRNA acts as an enhancer of translation of the *lacZ* gene in *Escherichia coli*. J Biol Chem. 1989;264:16973–6.

[13] Blattner FR, Plunkett G, Bloch CA, Perna NT, Burland V, Riley M, et al. The complete genome sequence of *Escherichia coli* K-12. Science. 1997;277:1453–62.

[14] Scholtissek S, Grosse F. A cloning cartridge of lambda t_o_ terminator. Nucleic Acids Res. 1987;15:3185.

[15] Dunn JJ, Studier FW, Gottesman M. Complete nucleotide sequence of bacteriophage T7 DNA and the locations of T7 genetic elements. J Mol Biol. 1983;166:477–535.
